# Genome-resolved analysis of traditional fermented biofertilizers as scalable solutions for soil restoration

**DOI:** 10.3389/fmicb.2025.1725475

**Published:** 2025-12-29

**Authors:** Abhishek Walia, Ramganesh Selvarajan, Henry Joseph Oduor Ogola, Rakesh Chauhan, Jyoti Bala, Shailender Kumar Verma, Rameshwar Kumar

**Affiliations:** 1 Department of Microbiology, College of Basic Sciences, CSK Himachal Pradesh Krishi Vishvavidyalaya, Palampur, HP, India; 2 Department of Environmental Sciences (DES), College of Agriculture and Environmental Sciences (CAES), University of South Africa (UNISA), Florida Campus, Roodepoort, South Africa; 3 Department of Biochemistry, J.J College of Arts and Science (Autonomous), Pudukkottai, India; 4 Department of Organic Agriculture and Natural Farming, College of Agriculture, CSK Himachal Pradesh Krishi Vishvavidyalaya, Palampur, HP, India; 5 Department of Environmental Studies, University of Delhi, Delhi, India

**Keywords:** biofertilizer, *Jeevamrit*, metagenomics, microbial consortium, nutrient cycling, soil restoration, sustainable agriculture

## Abstract

Soil degradation threatens global food security by eroding nutrient reserves and biological resilience. Microbial solutions that regenerate soil fertility through ecological processes offer a sustainable alternative to chemical intensification, yet lack mechanistic validation linking genomic potential to field performance. Fermented microbial consortia, naturally assembled through traditional practices worldwide, represent promising but underexplored technologies for biological soil restoration. Here, we integrate shotgun metagenomics, metagenome-assembled genome (MAG) reconstruction, and two-season field trials to evaluate *Jeevamrit*, a cattle-derived fermented biofertilizer widely used across South Asia, as a model system for understanding microbial-mediated soil restoration. Metagenomic profiling revealed that *Jeevamrit* fermentation of cattle dung and urine produces a functionally rich microbial consortium dominated by Firmicutes, Proteobacteria, Actinobacteria, and Bacteroidetes. Thirty high-quality MAGs encoded genes for nitrogen fixation (*nifHDK*), phosphate solubilization (*phoA, pstS*), potassium transport (*trkA, phoR*), siderophore biosynthesis, and phytohormone production (*trpA, miaB*), alongside enriched CAZymes (GH13, PL1) and biosynthetic clusters (NRPS, PKS, terpenes) supporting nutrient turnover and rhizosphere signaling. Field application in severely degraded Himalayan rice soils substantially improved soil health relative to controls: soil organic carbon increased from 0.53%–0.68% to 0.76%–1.04% (up to 96% increase), microbial biomass carbon rose from ~72 mg C kg^−1^ to 186–282 mg C kg^−1^ (159% increase), available phosphorus increased 39.5%, and grain yield improved 74%, while pH and electrical conductivity remained stable. Principal component analysis confirmed that SOC, microbial biomass, and nutrient availability drove treatment differentiation, corroborating genomic predictions. This genome-to-field framework establishes fermented microbial consortia as multifunctional solutions that restore soil fertility through ecological intensification rather than chemical supplementation. By demonstrating that traditional farmer innovations can be genomically validated and mechanistically understood, this work provides a replicable model for scaling nature-based, low-cost soil restoration technologies to address global agricultural sustainability challenges.

## 1 Introduction

Soil degradation is a pervasive global challenge that threatens agricultural productivity, food security, and ecosystem stability. Approximately one-third of the world’s arable land is degraded due to intensive cultivation, chemical fertilizer dependence, and the erosion of soil organic matter and microbial diversity (Lal, 2015; Smith et al., 2024). These losses impair nutrient cycling, carbon sequestration, and the soil’s inherent biological buffering capacity, ultimately constraining crop yields and resilience under climate variability, affecting over 2 billion smallholder farmers who depend on degraded soils for their livelihoods (Smith et al., 2024). Conventional remediation strategies, primarily chemical fertilizers, provide transient productivity gains but fail to restore the biological integrity required for sustainable fertility. Consequently, there is growing recognition that soil restoration must be biologically driven, emphasizing microbial ecological processes that rebuild nutrient turnover, organic matter stabilization, and plant–microbe symbioses (Chaparro et al., 2012; Compant et al., 2019).

Microbial communities form the living engine of soils, orchestrating elemental cycling and ecosystem function through diverse metabolic pathways. These microbial networks regulate key processes including nitrogen fixation, phosphorus solubilization, mineral weathering, and decomposition of organic residues (Nannipieri et al., 2011). Decline in microbial abundance and diversity is one of the earliest and most severe consequences of soil degradation, particularly in acidic and nutrient-depleted tropical and subtropical soils, a category encompassing over 1.5 billion hectares globally across South Asia, Sub-Saharan Africa, Southeast Asia, and Latin America (Smith et al., 2024). The Himalayan foothills exemplify these conditions, where intensive agriculture has depleted both soil organic matter and microbial diversity. Restoring microbial function in such systems is therefore central to re-establishing soil fertility and long-term productivity. While synthetic inoculants and engineered consortia have been explored as biofertilizers, their efficacy often declines under field conditions due to ecological incompatibility and limited adaptability (Lal, 2015). This is particularly relevant to smallholder farmers in low-income regions, where synthetic inoculants are often prohibitively expensive or require cold-chain infrastructure unavailable in rural settings. This limitation has prompted interest in naturally fermented microbial consortia, which harbor ecologically co-adapted communities capable of thriving in dynamic soil environments.

Globally, naturally fermented organic fertilizers such as bokashi (Japan), biol (Andean South America), and effective microorganisms (EM) solutions have been promoted as low-cost strategies to improve soil structure, microbial activity, and resilience (Abo-Sido et al., 2021; O’Neill and Ramos-Abensur, 2022; Prisa and Jamal, 2025). Among such systems, India’s *Jeevamrit*, a farmer-developed fermented organic formulation, represents one of the most widely adopted farmer-driven biofertilizers globally, with an estimated 5 million+ farmers across South Asia applying it on over 10 million hectares (Kumar et al., 2023). Prepared from locally available cattle dung, urine, pulse flour, jaggery, and soil, ingredients accessible to smallholder farmers across tropical and subtropical regions, *Jeevamrit* exemplifies how traditional fermentation practices can be standardized for broad uptake while retaining adaptability to local resource availability. While EM and bokashi have achieved commercial distribution in Asia and Latin America, *Jeevamrit’s* remarkable spread has occurred almost entirely through farmer-to-farmer knowledge transfer, requiring minimal external inputs or technical infrastructure (Kumar et al., 2023). This grassroots scalability, combined with preparation costs estimated at <$5 USD per hectare per application, distinguishes it as a model for resource-constrained agricultural settings globally. It is hypothesized that such fermented organic formulations enhance soil health and crop performance through their living microbial consortia, rather than through direct nutrient supplementation (Chakraborty et al., 2025). Recent studies have reported improvements in soil enzymatic activity, microbial biomass, and nutrient availability following *Jeevamrit* application (Duraivadivel et al., 2022; Saharan et al., 2023; Gurjar et al., 2024; Sharma et al., 2024; Mukherjee et al., 2025). However, despite its increasing adoption, the microbial mechanisms underlying these benefits remain poorly understood. Most previous studies have emphasized chemical or agronomic aspects, lacking genome-level insights into the microbial composition, functional potential, and soil restoration mechanisms of *Jeevamrit.*

A mechanistic understanding of *Jeevamrit*’s efficacy requires bridging genome-resolved microbial ecology with field-scale soil restoration outcomes. Advances in metagenomic tools now allow the reconstruction of metagenome-assembled genomes (MAGs), providing a detailed view of metabolic networks and gene functions within complex microbial consortia (Mirete et al., 2025). While fermented biofertilizers share the principle of microbial-mediated soil enhancement, their mechanistic pathways differ substantially based on substrate composition, fermentation conditions, and microbial assembly processes. Bokashi, originating from Japan, relies on lactic acid bacteria-dominated consortia fermenting mixed organic matter under anaerobic conditions, producing organic acids that accelerate decomposition but with limited diazotrophic capacity (Pandit et al., 2020). Recent genomic investigations of bokashi have revealed enrichment of *Lactobacillus* and *Bacillus* species with carbohydrate-active enzymes but documented minimal representation of assimilatory nitrate reduction and phosphate-solubilizing gene clusters compared to synthetic inoculants (Abo-Sido et al., 2021). EM solutions, though widely commercialized, utilize defined inoculants of photosynthetic bacteria, lactic acid bacteria, and yeasts, which may lack the ecological co-adaptation necessary for resilience in degraded field soils (Li et al., 2024). Andean biol systems ferment plant residues and animal manures anaerobically, enriching nitrogen-processing guilds, yet genome-resolved characterization of their functional networks remains limited (O’Neill and Ramos-Abensur, 2022). Preliminary evidence suggests cattle-based fermentations may harbor broader functional redundancy due to herbivore gut microbiome contributions (Ziganshin et al., 2016; Chakraborty et al., 2025), yet this hypothesis lacks genome-to-field validation. *Jeevamrit* distinguishes itself through three key features: (i) co-fermentation of cattle dung and urine, integrating complementary microbial guilds, fibrolytic anaerobes from dung and nitrogen-cycling aerobes from urine; (ii) inclusion of pulse flour and jaggery as metabolic substrates that select for multifunctional taxa; and (iii) grassroots scalability without cold-chain requirements or proprietary inoculants. However, mechanistic understanding of how these design features translate into functional gene enrichment, field-scale nutrient cycling, and sustained soil recovery remains absent from the literature.

This knowledge gap has critical consequences: without mechanistic evidence, traditional practices, even those adopted by millions of farmers, remain excluded from climate finance mechanisms (e.g., soil carbon sequestration credits), international regulatory frameworks, and evidence-based agricultural development programs. Applying genome-resolved approaches to traditional fermented systems can reveal the genetic basis of nutrient transformation, organic matter turnover, and resilience, transforming empirical practices into scientifically validated, scalable microbial technologies (Bargaz et al., 2018). In the broader context of global soil degradation, *Jeevamrit* thus provides a unique model system for exploring how microbial ecological intensification can regenerate soil function through community-level processes, while establishing a replicable validation framework for integrating farmer-derived innovations into global sustainable agriculture portfolios.

The present study aimed to elucidate the microbial and functional mechanisms through which the fermented microbial consortium *Jeevamrit* restores soil fertility and crop productivity in degraded, rice-based agroecosystems. Specifically, we (i) characterized the taxonomic and functional composition of the *Jeevamrit* microbiome using shotgun metagenomics and MAG reconstruction; (ii) identified microbial genes and pathways involved in nutrient cycling, organic matter transformation, and plant growth promotion; and (iii) validated these genomic predictions through two-season field trials assessing soil biological recovery and yield performance. The study was conducted in the acidic, nutrient-depleted soils of the Himalayan mid-hills, conditions affecting over 1.5 billion hectares globally, representing a model environment for evaluating microbial restoration in degraded systems with direct applicability to analogous soil types across tropical and subtropical zones worldwide.

This study establishes *Jeevamrit* as a model system for genome-to-field validation of farmer-innovated biofertilizers, with direct applicability to fermented organic fertilizer systems worldwide. The acidic, nitrogen-depleted soils of the Himalayan mid-hills represent conditions tropical and subtropical zones (Smith et al., 2024), making findings directly transferable to degraded systems in Sub-Saharan Africa, Southeast Asia, Latin America, and Pacific Island nations. By demonstrating that farmer-developed fermentation processes yield genomically characterizable, functionally predictable, and agronomically effective microbial consortia, we provide a replicable methodology for validating traditional soil restoration practices. Beyond mechanistic insights, this work advances practical pathways for scaling nature-based, low-cost agricultural technologies. The integration of indigenous agroecological knowledge with genome-resolved microbial ecology establishes a framework for developing standardized yet locally adaptable biofertilizers that support regenerative agriculture, enhance climate resilience through soil carbon sequestration, and improve food security.

## 2 Materials and methods

### 2.1 Sample collection

Fresh cow dung and urine samples were collected from three lactating cattle breeds: *Himachal Pahari* (PL), Jersey (JL), and Sahiwal (SL). *Himachal Pahari* samples were obtained from the Dairy Unit of the Department of Organic Agriculture and Natural Farming, College of Agriculture, CSK Himachal Pradesh Krishi Vishvavidyalaya (CSK HPKV), Palampur, Himachal Pradesh, India (32.110°N, 76.543°E; elevation ~1,220 m a.s.l.). Jersey and Sahiwal samples were sourced from the Dairy Farm of the Department of Livestock Management, College of Veterinary and Animal Sciences, CSK HPKV, Palampur. For each breed, three independent biological replicates were collected aseptically, transported under cold-chain conditions (4 °C ± 2 °C), and processed immediately.

### 2.2 Preparation of fermented Jeevamrit bioformulations

*Jeevamrit* was prepared following a standardized protocol adapted from Shraddha et al. (2023), with slight modifications ensuring uniformity across breeds. Briefly, each batch combined 1.0 kg of fresh dung and 1.0 L of fresh urine from the same animal with 200 g of jaggery, 200 g of gram flour (*Cicer arietinum*), and 100 g of native soil (collected from the top 0–10 cm of a cultivated organic field). Twenty liters of potable water were added to the mixture in a high-density polyethylene fermentation container (25 L capacity) previously sanitized with 70% ethanol.

The slurry was manually stirred three times daily using a flame-sterilized wooden stick, alternating clockwise and anticlockwise directions, to ensure homogeneous mixing and aeration. Fermentation was carried out under shaded ambient conditions (25 °C–32 °C; relative humidity 60%–75%). *Jeevamrit* was fermented for 5–7 days until the system reached maturity, indicated by stable pH and gas production cessation. For all downstream experiments, including metagenomic analysis and field trials, the 7-day fermented product was used. For each cattle breed, three independent *Jeevamrit* fermentations were prepared in parallel, corresponding to the three biological replicates of dung and urine. Immediately after fermentation, aliquots (50 mL) were collected aseptically for metagenomic DNA extraction and functional analyses.

### 2.3 DNA extraction, library preparation, and shotgun metagenomic sequencing

Total genomic DNA was extracted from 1.0 g of homogenized solid matrices (*Jeevamrit* sediment and dung), and from 200 mL urine samples passed through sterile 0.22 μm pore-size membrane filters (Millipore, USA). DNA was extracted using the DNeasy PowerSoil Pro Kit (Qiagen, Hilden, Germany). Each extraction batch included a sterile media negative control, which was processed through DNA extraction, library preparation, and sequencing workflows to assess background contamination.

DNA quality was assessed using Qubit 4.0 fluorometry and NanoDrop spectrophotometry, while integrity was verified on 1.2% agarose gel electrophoresis. Sequencing libraries were prepared from equimolar pools of the three biological replicates per substrate (dung, urine, *Jeevamrit*) and cattle breed (PL, JL, SL), resulting in nine composite DNA samples. Prior to pooling, replicate-level similarity was confirmed through taxonomic profiling, with Bray–Curtis dissimilarity values <0.12 across replicates, indicating high compositional consistency. Pooling was therefore deemed appropriate to (i) construct representative substrate-level metagenomes and (ii) ensure sufficient sequencing depth for high-quality MAG reconstruction.

Libraries were prepared using the Twist NGS Library Preparation Kit (Twist Biosciences, USA) and quality-checked on an Agilent 2,100 Bioanalyzer. Sequencing was conducted on the Illumina NovaSeq 6000 platform (2 × 150 bp). All raw sequence data have been deposited in the NCBI Sequence Read Archive under BioProject ID PRJNA1162843, with individual SRA accessions SRR30724627 to SRR30724635.

### 2.4 Bioinformatic analysis

#### 2.4.1 Metagenomic workflow and MAG reconstruction

Reads were processed on the DOE KBase^1^ (Arkin et al., 2018). Quality control included FastQC (v0.11.9) (Andrews et al., 2010) inspection and Trimmomatic (Bolger et al., 2014) trimming and filtering, with the following parameters: LEADING:20, TRAILING:20, SLIDINGWINDOW:4:20, and MINLEN:50. Reads from negative controls were processed alongside biological samples to verify absence of contaminants. Read-based taxonomic classification was performed using Kaiju (v1.8.2) in Greedy mode against the NCBI nr reference database (Menzel et al., 2016).

*De novo* assembly was performed using Megahit v1.2.9 (Li et al., 2016), with default parameters. To increase robustness of MAG recovery, three binning tools, MetaBAT2 v2.15 (Kang et al., 2019), MaxBin2 v2.2.7 (Wu et al., 2016), and CONCOCT v1.1.0 (Alneberg et al., 2014), were applied independently. Output bins were refined and dereplicated using DAS Tool v1.1.6 (Sieber et al., 2018) and dRep v2.0.0 (Olm et al., 2017) to generate a nonredundant, consensus MAG set. MAG completeness and contamination were evaluated using CheckM v1.2.0 (Parks et al., 2015), and only MAGs with meeting a minimum of ≥70% completeness and <10% contamination were retained. Taxonomic classification of the MAGs was performed using GTDB-Tk v2.3.0 (Chaumeil et al., 2022) against the GTDB release r214. The combination of multi-tool binning followed by dereplication provided an inherent sensitivity assessment by comparing binning congruence across MetaBAT2, MaxBin2, and CONCOCT. Only bins consistently supported across tools or selected as highest-quality representatives by DAS Tool/dRep were retained, minimizing tool-specific biases. All high-quality MAGs recovered in this study have been deposited in the Figshare repository.^2^

The mean depth of sequencing coverage for each metagenome was assessed using CoverM v0.3.2 (default parameters).^3^ Furthermore, to estimate the relative abundance of the recovered MAGs within each metagenomic sample, sequencing reads were mapped to a combined reference file of all 30 MAGs, using CoverM with its default configuration.

#### 2.4.2 Functional annotation

The initial analysis utilized the Distilled and Refined Annotation of Metabolism (DRAM v1.4.4) tool (Shaffer et al., 2020) to conduct a curated assessment of plant growth-promoting traits (nitrogen fixation, IAA biosynthesis, ACC deaminase, phosphate/potassium solubilization and transport) and carbohydrate-active enzymes (CAZymes). Complementing this, a second pipeline, PGPg_finder v1.1.0 (Pellegrinetti et al., 2024), was used to predict PGP traits. This involved predicting open reading frames with Prodigal (Hyatt et al., 2010) and performing sequence alignments using DIAMOND’s blastx function (Buchfink et al., 2021), against the specialized PLaBAse–PGPT-db database for assessing the plant growth-promoting potential of plant-associated bacteria (Patz et al., 2021).

#### 2.4.3 Biosynthetic gene clusters and secondary metabolite potential

To assess the potential for secondary metabolite biosynthesis, dereplicated MAGs were screened with antiSMASH v7.0 (Blin et al., 2023), using default parameters and “relaxed” strictness settings to detect both canonical and novel biosynthetic architectures. Predicted BGCs were annotated for core biosynthetic genes, cluster boundaries, and putative product classes, and were compared against reference clusters in the MIBiG repository (Terlouw et al., 2023).

### 2.5 Field trial validation

Field experiments were conducted over two consecutive growing seasons (Kharif 2022 and Rabi 2023–24) at the Zero Budget Natural Farm (ZBNF), Department of Organic Agriculture and Natural Farming, CSK Himachal Pradesh Krishi Vishvavidyalaya, Palampur. The site is located in the Palampur valley, Kangra district, Himachal Pradesh (32°6′N latitude, 76°3′E longitude) at 1290.8 m above mean sea level, within the mid-hill sub-humid zone of the North Western Himalayas. The region receives approximately 2,100 mm average annual rainfall, predominantly during the monsoon season, with occasional cyclonic showers in winter (Bahadori and Tofighi, 2017).

#### 2.5.1 Site characterization and baseline soil status

The experimental soil was silty clay loam in texture, acidic in reaction (pH < 5.76), with medium organic carbon (SOC = 0.60% ± 0.10%) available phosphorus (15.8 kg ha^−1^) and potassium (92.6 kg ha^−1^), but low available nitrogen (170.5 kg ha^−1^). Continuous potato cultivation in preceding years had caused significant soil degradation, particularly nitrogen depletion and loss of biological activity. Microbial biomass carbon (MBC) at baseline was 88.5 ± 4.2 mg C kg^−1^, and dehydrogenase activity (DHA) was 89.3 ± 3.1 mg TPF g^−1^ h^−1^, both indicative of severely depleted soil biological function. This degraded condition provided an ideal scenario to evaluate *Jeevamrit*’s soil restoration potential.

#### 2.5.2 Experimental design and treatment structure

The trial followed a Randomized Block Design (RBD) with three replications using two rice varieties, HPR-1068 (V1) and HPR-2880 (V2), across eight treatments: T1 (V1 + *Jeevamrit* at 14-day intervals), T2 (V1 + *Jeevamrit* at 21-day intervals), T3 (V1 + *Jeevamrit* at 28-day intervals), T4 (V2 + *Jeevamrit* at 14-day intervals), T5 (V2 + *Jeevamrit* at 21-day intervals), T6 (V2 + *Jeevamrit* at 28-day intervals), T7 (V1 control, no *Jeevamrit*), and T8 (V2 control, no *Jeevamrit*). In total, 24 plots (8 treatments × 3 replications) were established. Each gross plot measured 3.0 m × 3.0 m (9.0 m^2^), with a net plot size of 2.40 m × 2.80 m (6.72 m^2^) maintained at an optimal plant density of 150 plants m^−2^. Treatments were randomly assigned within each block to minimize experimental error. This factorial design (2 varieties × 3 application frequencies + 2 controls) enabled assessment of both varietal response and optimal *Jeevamrit* application frequency. The same plots were maintained across both growing seasons to track cumulative treatment effects.

#### 2.5.3 Crop establishment and *Jeevamrit* application protocol

Twenty-one-day-old rice seedlings were transplanted at 20 cm × 15 cm spacing in the third week of June 2022 (Kharif season) and November 2023 (dry/Rabi season). *Jeevamrit* was prepared fresh before each application following the protocol described in Section 2.2, and applied as a soil drench at 10% v/v dilution in water (application rate: 500 L ha^−1^ per application). Applications commenced 7 days after transplanting and continued at treatment-specific intervals (14, 21, or 28 days) until 15 days before physiological maturity. Control plots received equivalent volumes of water without *Jeevamrit*. During the Kharif (monsoon) season, plots were maintained under flooded conditions (5 ± 2 cm standing water) with natural monsoon rainfall supplemented by irrigation as needed. During the Rabi season, irrigation was scheduled at 3–4-day intervals to compensate for reduced winter precipitation and maintain adequate soil moisture. Standard agronomic practices, including manual weeding and pest monitoring, were uniform across all treatments. No synthetic fertilizers, pesticides, or other external inputs were applied throughout the experimental period.

#### 2.5.4 Harvest and sampling procedures

At physiological maturity (115–120 days after transplanting), grain yield was determined from the net plot area after sun-drying to 14% moisture content and expressed as quintals per hectare (q ha^−1^). Immediately following harvest, composite soil samples were collected from the 0–15 cm depth using a sterilized soil auger from five random locations per plot, thoroughly mixed, and divided into two subsamples. One subsample was air-dried, sieved (2 mm), and used for chemical analysis (soil organic carbon, pH, electrical conductivity, available N, P, and K). The second subsample was stored at 4 °C and analyzed within 48 h for biological parameters (microbial biomass carbon, dehydrogenase activity) and microbiological enumeration (general bacterial count, phosphate-solubilizing bacteria, nitrogen-fixing bacteria).

#### 2.5.5 Soil chemical and biological analyses

Soil organic carbon (SOC) was determined by the Walkley-Black wet oxidation method (Bahadori and Tofighi, 2017). Soil pH and electrical conductivity (EC) were measured in 1:2.5 soil:water suspensions using a digital pH meter and conductivity meter, respectively. Available nitrogen was estimated by the alkaline permanganate method (Stanford, 1978), available phosphorus by the Olsen method (Iatrou et al., 2014), and available potassium by flame photometry after extraction with neutral normal ammonium acetate (Asch et al., 2022). Microbial biomass carbon (MBC) was quantified using the chloroform fumigation-extraction method (Setia et al., 2012), with a KEC factor of 0.38 for conversion. Dehydrogenase activity (DHA) was measured using the triphenyl tetrazolium chloride (TTC) reduction method (Wolińska and Stępniewska, 2012), with activity expressed as mg triphenyl formazan (TPF) g^−1^ soil h^−1^.

#### 2.5.6 Microbiological enumeration

General bacterial counts were determined by serial dilution plating on nutrient agar, with incubation at 28 °C ± 2 °C for 48–72 h. Phosphate-solubilizing bacteria (PSB) were enumerated on Pikovskaya’s medium containing tricalcium phosphate, and colonies showing clear zones of solubilization were counted after 5–7 days of incubation. Nitrogen-fixing bacteria were enumerated on nitrogen-free Jensen’s medium, with incubation for 7–10 days at 28 °C± 2 °C. Actinomycetes were isolated and enumerated using Actinomycetes Isolation Agar (AIA; HiMedia, India). All microbiological counts were expressed as colony-forming units (CFU) per gram of oven-dry soil.

### 2.6 Statistical analyses and visualization

All data wrangling and statistical analyses were performed in R v4.3.1 using tidyverse, phyloseq, vegan, dplyr, and ggpubr. Prior to all analyses, data were assessed for normality using Shapiro–Wilk tests and for homogeneity of variance using Levene’s tests. Variables violating normality assumptions (*p* < 0.05) were log-transformed or square-root transformed as appropriate. Outliers were identified using the interquartile range (IQR) method (values > Q3 + 1.5 × IQR or < Q1–1.5 × IQR) and examined for biological validity; confirmed outliers resulting from technical error were excluded.

Alpha diversity indices (Shannon, Simpson, Chao1, ACE) were calculated from rarefied taxonomic profiles (95,600 reads per sample, representing the minimum library size) to ensure equal sampling depth. Beta diversity was evaluated using Bray-Curtis dissimilarities on Hellinger-transformed relative abundance data to reduce the influence of dominant taxa. Community composition was visualized via principal coordinates analysis (PCoA). Permutational multivariate analysis of variance (PERMANOVA) was performed using the adonis2 function (vegan package) with 9,999 permutations to test for significant differences across substrates and cattle breeds. Effect sizes were reported as *R*^2^ values representing the proportion of variance explained by each factor. Assumptions of multivariate homogeneity of dispersions were verified using betadisper and permutest functions (permutations = 999).

Core microbiota overlaps were explored through UpSet plots using UpSetR r package, resolving substrate-specific and shared genera. Differential abundance at the metagenome level was assessed using Linear Discriminant Analysis Effect Size (LEfSe) (Segata et al., 2011) and Random Forest (RF) classification modeling via the rfpermute package (Archer and Archer, 2016). LEfSe identified significantly enriched taxa (LDA > 2.0), while RF highlighted taxa critical for substrate discrimination, with model accuracy evaluated through 10-fold cross-validation and out-of-bag error estimation. Feature importance was assessed using mean decrease in Gini impurity.

Taxonomic distributions of MAGs were represented with Sankey diagrams, depicting lineage flows. Functional profiles, including plant growth-promoting traits, CAZy families and BGCs, were analyzed at metagenome and MAG levels and visualized as *z*-score–scaled heatmaps with hierarchical clustering (Euclidean distance, Ward’s linkage method; ComplexHeatmap package) to reveal functional pattern similarities.

Data collected from field trials were subjected to repeated measures analysis of variance (ANOVA) using the lme4 package, with treatment as fixed effect and block as random effect to account for spatial heterogeneity. Residuals were examined for normality (Shapiro–Wilk test) and homoscedasticity (Levene’s test). Soil organic carbon (SOC) and microbial biomass carbon (MBC) data were log-transformed to meet normality assumptions. Treatment means were compared using Duncan’s Multiple Range Test (DMRT) at *α* = 0.05, implemented via the agricolae package. Percentage increases over controls were calculated as: [(Treatment mean − Control mean) / Control mean] × 100. Confidence intervals (95% CI) for percentage changes were estimated using parametric bootstrap resampling (10,000 iterations) via the boot package, accounting for within-treatment variance. PCA was conducted on standardized (*z*-score transformed) soil parameters using the prcomp function. Prior to PCA, multivariate outliers were assessed using Mahalanobis distance (*χ*^2^ critical value at *p* < 0.001); no outliers were detected. The Kaiser–Meyer–Olkin (KMO) measure of sampling adequacy (0.847) and Bartlett’s test of sphericity (*p* < 0.001) confirmed data suitability for PCA. Component loadings > |0.40| were considered meaningful contributors to each principal component.

All figures were generated in ggplot2 and optimized for publication quality. Statistical significance was set at *p* < 0.05 unless otherwise specified, and all *p*-values were adjusted for multiple comparisons using the Benjamini–Hochberg false discovery rate (FDR) procedure where applicable.

## 3 Results

### 3.1 Global sequencing output and microbial diversity across substrates

Metagenomic sequencing generated a total of 13.3–27.4 million raw reads per sample, with consistently high read quality (>95.9%) (Table 1). Across all datasets, GC content varied between 37.9% and 54.3%, reflecting compositional differences in microbial communities. Taxonomic assignment via Kaiju demonstrated higher classification rates in urine (73.4%–85.8%) relative to dung (38.1%–40.3%) and *Jeevamrit* (37.5%–46.8%), indicating greater representation of uncharacterized microbial taxa in the latter two substrates.

**Table 1:** Summary of global metagenomic sequencing output, assembly metrics, Kaiju-based taxonomic profiles, and alpha diversity indices for bacterial communities in urine, dung, and *Jeevamrit* from Sahiwal (SL), Himachal Pahari (PL), and Jersey (JL) cattle.

Category	Urine			Dung			*Jeevamrit*		
	SLU	PLU	JLU	SLD	PLD	JLD	SLJ	PLJ	JLJ
SRA Accession	SRR30724627	SRR30724630	SRR30724633	SRR30724629	SRR30724632	SRR30724635	SRR30724628	SRR30724631	SRR30724634
Raw reads	13.58M	15.44M	19.44M	26.24M	19.11M	19.68M	13.31M	16.73M	27.44M
Quality reads (%)	96.0	96.4	96.4	96.2	96.0	95.9	96.9	96.8	96.0
Reads mapped to contigs (%)	65.1	64.8	76.9	67.9	78.9	75.3	71.8	65.8	81.2
Reads mapped to CDS (%)	55.7	51.2	62.1	54.1	65.4	68.9	53.3	56.7	70.9
GC content (%)	47.6	37.9	44.2	44.3	54.3	47.6	44.7	45.1	45.6
Kaiju classification (%)	85.8	81.1	73.4	38.1	40.3	39.9	46.8	45.2	37.5
Bacteria/Archaea	98.5	99.2	98.9	99.7	98.7	98.9	99.0	98.2	99.5
Fungi	0.1	0.2	0.4	0.01	0.6	0.8	0.2	1.0	0.1
Viruses	1.4	0.6	0.7	0.26	0.7	0.3	0.8	0.8	0.4
Alpha diversity indices									
Chao1	2,565	1,764	3,688	6,280	6,021	6,081	6,280	5,842	6,075
ACE	2,556	1,820	3,678	6,207	5,954	5,971	6,207	5,756	6,053
Shannon	2.66	1.23	3.81	5.31	5.16	5.20	5.31	4.80	5.23
Simpson	0.87	0.56	0.94	0.98	0.98	0.98	0.98	0.97	0.98
Coverage (%)	99.9	99.9	99.9	99.9	99.9	99.9	99.9	99.9	99.9

Bacteria and archaea dominated across all samples (>98%), while fungal and viral fractions were minor (<1.0%). Notably, dung and *Jeevamrit* derived from Himachal Pahari cattle showed comparatively higher fungal signals (0.6%–1.0%), suggesting subtle breed-linked influences on community structure. Viral sequences were proportionally enriched in urine (0.6%–1.4%).

Alpha diversity metrics revealed clear substrate-level differences. Urine communities were the least diverse, with lower richness (Chao1: 1,764–3,688) and evenness (Shannon: 1.23–3.81) compared to the other substrates. Dung samples harbored the richest and most even communities (Chao1: 6,021–6,280; Shannon: 5.16–5.31). *Jeevamrit* exhibited similarly high richness (Chao1: 5,842–6,280) and comparable evenness (Shannon: 4.80–5.31) to dung. Simpson Index values supported this pattern, indicating reduced stability and evenness in urine (Simpson: 0.56–0.94), while dung and *Jeevamrit* communities were consistently more even (Simpson: 0.97–0.98). Sequencing coverage was uniformly high (99.9%) across all samples, ensuring robust representation of microbial diversity.

### 3.2 Substrate type drives bacterial community structure

**Figure 1:**
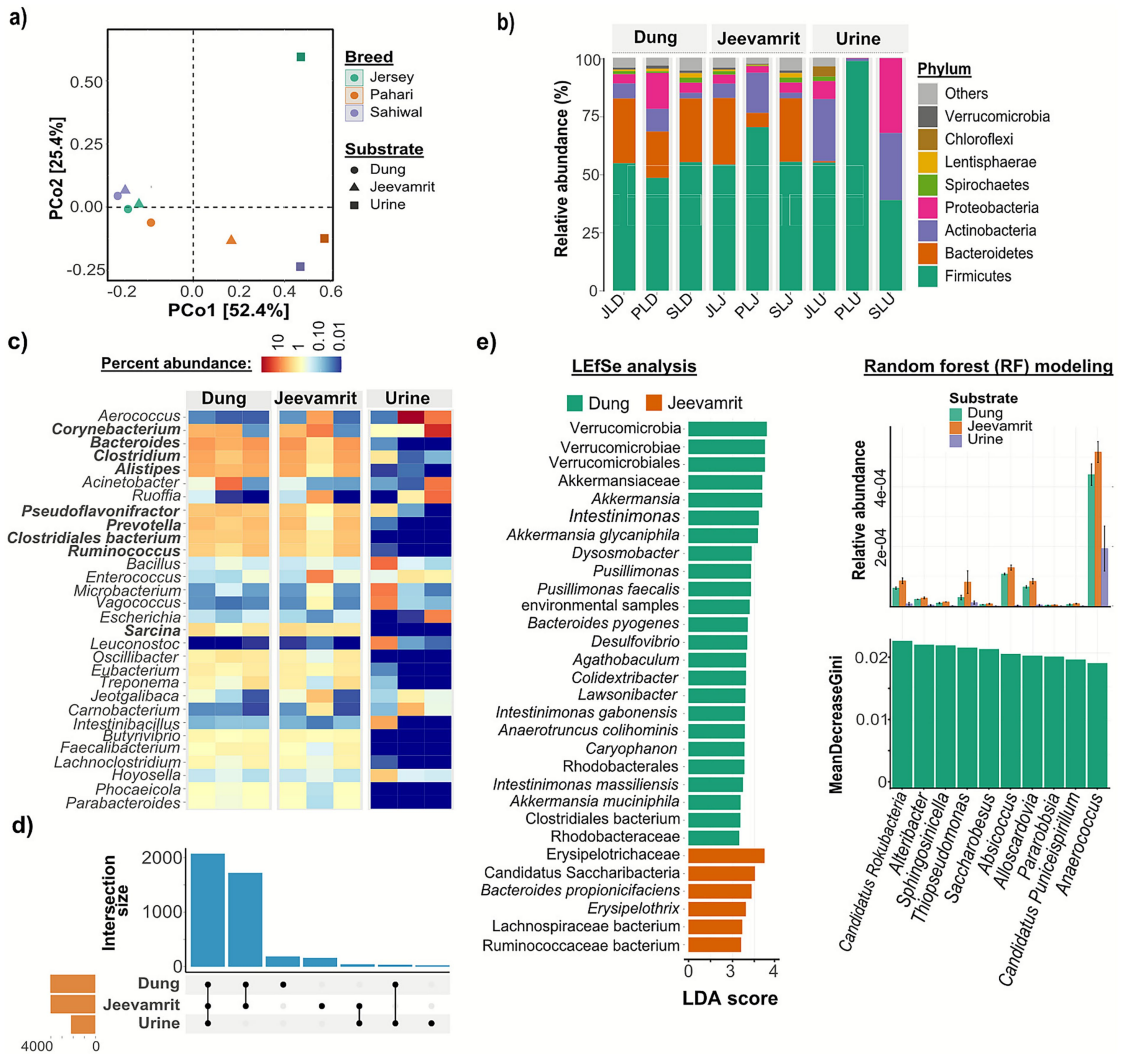
Substrate-driven structuring of bacterial communities in *Jeevamrit* and source inputs (dung, urine). **(a)** PCoA based on Bray–Curtis distances showing distinct clustering by substrate, with minimal influence of cattle breed. **(b)** Relative abundance of bacterial phyla highlighting substrate-specific signatures: dung dominated by Firmicutes (35%–48%) and Bacteroidetes (22%–30%), urine showing variable dominance across samples, with Proteobacteria enriched in SLU and Firmicutes predominant in the remaining samples, and *Jeevamrit* exhibiting a hybrid profile retaining dung-associated Firmicutes and Bacteroidetes while incorporating urine-derived Proteobacteria. **(c)** Heatmap of the top 30 bacterial genera revealing clear substrate-specific enrichment patterns. **(d)** UpSet plot depicting unique and shared core genera, with *Jeevamrit* retaining contributions from both dung and urine. **(e)** LEfSe and random forest analyses identifying discriminant bacterial taxa, with fibrolytic *Ruminococcus* and *Clostridium* enriched in dung, nitrogen-cycling *Pseudomonas* in urine, and multifunctional genera such as *Bacillus* and *Azotobacter* enriched in *Jeevamrit*.

Beta diversity analysis based on Bray–Curtis dissimilarities (Figure 1a) showed that bacterial community composition was strongly structured by substrate (PERMANOVA, *F* = 6.54, *R^2^* = 0.2345, *p* = 0.0153), but not by cattle breed (*F* = 0.98, *R^2^* = 0.0261, *p* = 0.6110). Fermentation attenuated breed-specific signatures, as *Jeevamrit* and dung clustered closely, while urine formed distinct groups reflecting its lower microbial complexity (Antaliya et al., 2025). Hierarchical clustering (Supplementary Figure S1a) further highlighted substrate specificity, with notable proximity between Jersey dung (JLD) and its derived *Jeevamrit* (JLJ), underscoring dung as a primary inoculum source in fermentation.

Taxonomic profiling revealed clear substrate-dependent bacterial signatures (Figures 1b–d). Dung harbored a taxonomically rich microbiota dominated by Firmicutes (35%–48%), Bacteroidetes (22%–30%), Spirochetes (5%–8%), and Actinobacteria (3%–6%), consistent with herbivore gut ecosystems. *Jeevamrit* exhibited a hybrid profile, retaining dung-associated Firmicutes (38%–45%) and Bacteroidetes (20%–28%) while incorporating a distinct Proteobacteria fraction (15%–22%) traceable to urine inputs. At the class level, both dung and *Jeevamrit* were enriched in Clostridia, Bacteroidia, and Bacilli (Supplementary Figure S1b). Genus-level resolution reinforced these patterns (Figure 1c). Dung was enriched in obligate anaerobes and fibrolytic fermenters such as *Ruminococcus*, *Faecalibacterium*, *Clostridium*, *Prevotella*, *Treponema*, and *Lachnoclostridium*. In contrast, urine samples were dominated narrow set taxa belonging to phylum Firmicutes (37%–98%), Actinobacteria (0.2%–19%), and nitrogen-processing and stress-tolerant protoebacterial taxa, including *Escherichia*, *Microbacterium*, *Acinetobacter*, and *Aerococcus*. *Jeevamrit* uniquely integrated guilds from both inputs while selectively enriching facultative fermenters and sporulators such as *Bacillus*, *Sarcina*, *Leuconostoc*, and *Jeotgalibaca*, reflecting fermentation-driven community assembly and multifunctionality.

Core microbiome analysis (Figure 1d) revealed that *Jeevamrit* shares the majority of its bacterial genera with dung, confirming dung as the primary microbial seed bank, while urine contributed a narrower but functionally specialized set of taxa associated with nitrogen transformations. No breed-specific core taxa were identified, underscoring that substrate composition, rather than host genetics, governs microbial community assembly. To identify discriminant taxa, we applied LEfSe and Random Forest (RF) analyses (Figure 1e). LEfSe highlighted strong substrate-specific signatures: dung was enriched in fibrolytic and SCFA-producing anaerobes, including *Akkermansia* (Verrucomicrobiales), *Agathobacter*, *Colidextribacter*, *Anaerotruncus*, and multiple *Clostridiales*. In contrast, *Jeevamrit* was enriched in members of the *Ruminococcaceae* and *Lachnospiraceae* families, along with *Erysipelothrix* and *Candidatus Saccharibacteria*, reflecting functional shifts during fermentation. RF analysis corroborated these findings, identifying *Candidatus Rokubacteria*, *Alteribacter*, and *Sphingosinicella* as key classifiers. The overlap between LEfSe and RF (e.g., *Rokubacteria*, *Saccharibacteria*) strengthens their value as ecological biomarkers defining substrate-specific community assembly.

These results demonstrate that fermentation of cattle inputs into *Jeevamrit* enriches microbial richness and sustains diversity comparable to dung, while shifting community evenness. This supports the hypothesis that *Jeevamrit* acts as a functionally diverse microbial consortium. These genomic baselines provided the foundation for subsequent analyses of plant growth–promoting traits, metabolic capacity, and biosynthetic potential, both at the reads and MAG level, as well as validation of functional relevance for soil restoration in field trials.

### 3.3 Functional capacity for nitrogen cycling and plant growth promotion

#### 3.3.1 Nitrogen cycling pathways

**Figure 2:**
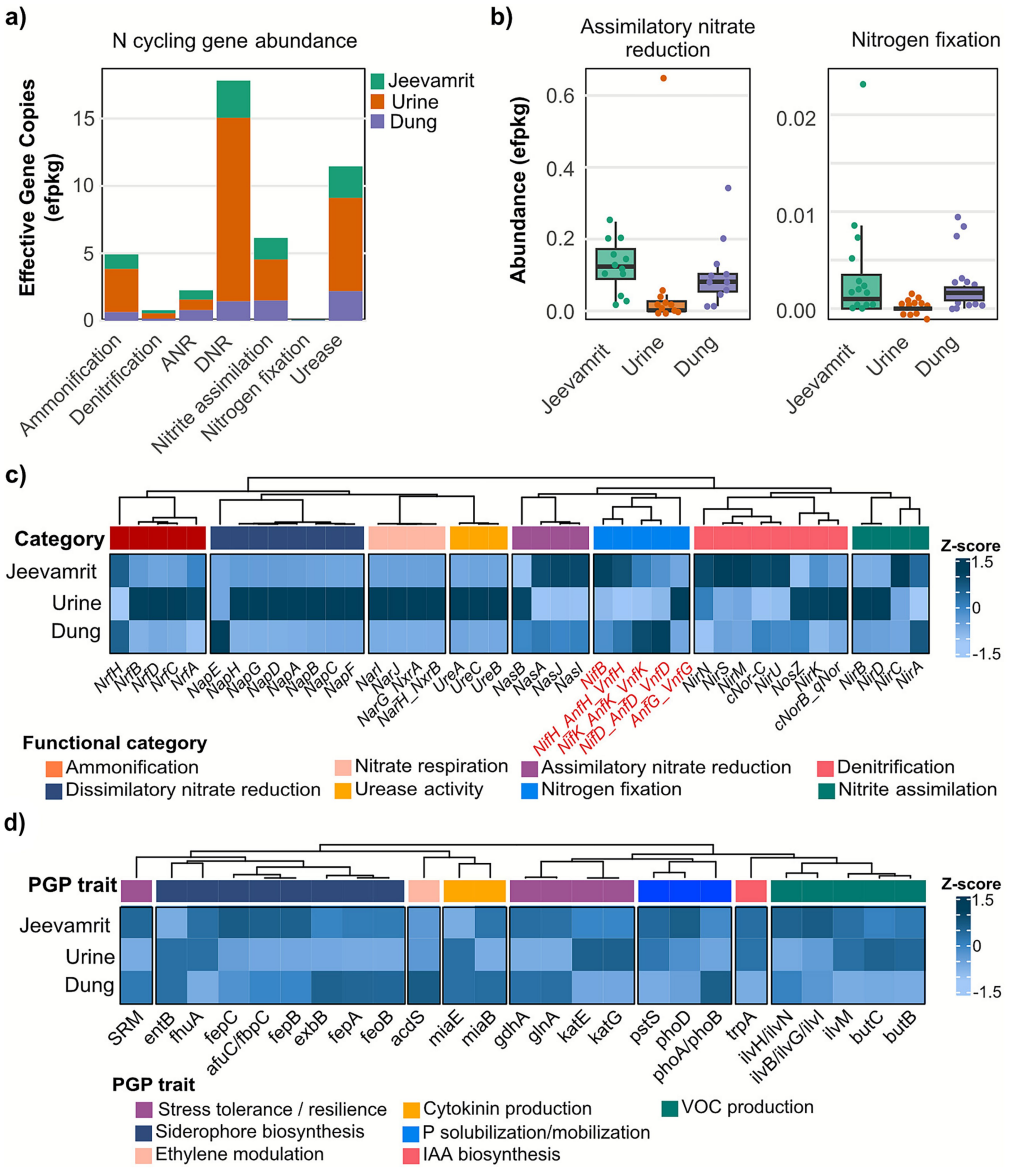
Nitrogen functional gene profiles and plant growth–promoting (PGP) potential across substrates. **(a)** Relative abundance (measured in efpkg: estimated fragments per kilobase of gene per gigabase of metagenome) of major nitrogen cycling functional categories in *Jeevamrit*, dung, and urine. *Jeevamrit*. **(b)** Kruskal–Wallis statistical comparison of nitrogen functional categories. Genes involved in nitrogen fixation, a key plant growth–promoting trait, are highlighted in red. **(c)** Heatmap displaying the abundance of individual nitrogen cycling genes across the three substrates, grouped by functional category. **(d)** Annotated heatmap of genes associated with PGP traits across substrates with genes are grouped according to biological. *Z*-score normalization was applied to emphasize relative differences across samples. Heatmaps are presented as complementary visual summaries of patterns already captured in panels **(a,b)**; hierarchical clustering was not applied because it did not yield additional interpretive value.

Metagenomic profiling revealed a broad distribution of nitrogen cycling genes spanning ammonification, urease activity, assimilatory and dissimilatory nitrate reduction, nitrite assimilation, denitrification, and nitrogen fixation (Figure 2a; Supplementary Table S1). Urine microbiomes were enriched in genes supporting rapid nitrogen turnover, including urease (*ureABC*), ammonia assimilation (*gdhA*), nitrite assimilation (*nirA*), and dissimilatory nitrate reduction (*napA, nirK*), consistent with their adaptation to a high-nitrogen, low-carbon environment. These specialized guilds promote short-term N release but offer limited buffering against leaching or gaseous loss.

In contrast, *Jeevamrit* exhibited significant enrichment of assimilatory nitrate reduction and nitrogen fixation pathways relative to dung and urine (Kruskal–Wallis, *p-adj* = 0.0057) (Figure 2b). Heatmap analyses (Figure 2c) confirmed elevated abundances of *nasA* (K00372), *nasB* (K00362), and *nirA* (K00366), catalyzing nitrate (NO₃^−^) reduction to ammonium (NH₄^+^), alongside diazotrophic markers *nifH, nifD, nifK*. These shifts suggest that fermentation enriches microbial consortia with the dual capacity to recycle existing N pools and introduce new bioavailable N, critical for rebuilding fertility in degraded soils.

Taxonomic assignment linked these functions to distinct lineages. Dung supported diverse nitrate/nitrite reducers (*Acinetobacter, Psychrobacter, Clostridium, Bacillus, Denitrobacterium*), forming a broad but substrate-limited redundant network. *Jeevamrit*, however, selectively enriched metabolically versatile taxa such as *Bradyrhizobium elkanii, Clostridium celatum, Bacillus megaterium,* and actinobacterial lineages (*Streptomyces, Nocardiopsis, Dietzia*), encoding *nif* genes, assimilatory nitrate reductases, and urease operons. These guilds has a potential to enhance both functional redundancy and stability, creating a self-reinforcing N cycle that supports long-term soil recovery. Urine microbiomes, though less diverse, contained stress-tolerant taxa (*Moraxella, Macrococcus, Kocuria, Corynebacterium*) contributing primarily urease and ammonia assimilation functions, acting as narrow specialists with limited ecological resilience. These findings suggest that fermentation-driven assembly in *Jeevamrit* reconciles specialization (urine-derived rapid turnover) with redundancy (dung-derived multifunctionality), while enriching unique diazotrophic lineages. This functional integration underpins the potential restoration of degraded soils through enhancement of soil biological resilience, stabilizing nitrogen flows, rebuilding fertility, and reducing dependence on synthetic inputs.

#### 3.3.2 Multifunctional PGP gene repertoires

Extending beyond nitrogen cycling, we profiled seven key plant growth–promoting (PGP) traits across substrates. The *z*-score normalized heatmap (Figure 2d) revealed distinct substrate-specific enrichments, with dung and *Jeevamrit* showing consistently higher PGP gene abundances compared to urine. The ACC deaminase gene (*acdS*), which hydrolyzes 1-aminocyclopropane-1-carboxylate (ACC) to lower stress-induced ethylene levels, was detected exclusively in dung, suggesting its reduction or loss during fermentation. While this points to a potential trade-off, *Jeevamrit* compensated through enrichment of other stress-alleviating traits. Notably, siderophore biosynthesis genes (*fepA, fepB, feoB, afuC/fbpC, exbB, fhuA*) were strongly enriched in both dung and *Jeevamrit*, enhancing microbial competition for iron and indirectly promoting plant nutrient uptake in low-fertility soils.

Phosphate solubilization genes showed substrate-specific patterns: *pstS* (high-affinity phosphate transporter) was significantly elevated in *Jeevamrit*, indicating improved phosphate acquisition under limiting conditions, whereas *phoA* and *phoB* (alkaline phosphatases) were more abundant in dung, reflecting differences in inorganic phosphate mobilization strategies. These complementary pathways suggest that *Jeevamrit* integrates dung-derived solubilizers with fermentation-enriched high-affinity transporters to sustain phosphorus availability during soil restoration.

Genes involved in VOC biosynthesis, notably from branched-chain amino acid and butanediol pathways (*ilvB/G/H/I/N, butB, butC*), were enriched in dung and *Jeevamrit*. While upstream VOC genes (*alsS, alsD*) were sporadically detected, the presence of downstream pathway genes suggests alternative biosynthetic routes. In contrast, auxin biosynthesis gene *trpA* (tryptophan pathway) was detected across all substrates, with higher abundance in *Jeevamrit* and urine, pointing to a generalized capacity for root elongation and branching. Cytokinin biosynthesis-related genes (*miaB, miaE*) were broadly distributed, with *miaB* notably enriched in dung. The polyamine biosynthesis gene *speE* (spermidine synthase), important for stress tolerance and plant development, was modestly represented, with slightly higher abundance *Jeevamrit,* suggesting a role in enhancing plant recovery under nutrient-limited conditions.

Mechanistically, these findings highlight that while dung serves as the primary reservoir of diverse PGP traits, fermentation into *Jeevamrit* selectively enriches high-affinity nutrient transporters, siderophore producers, and auxin-associated pathways. Together, these functions create a multifunctional microbial repertoire capable of restoring degraded soils by improving nutrient mobilization, buffering stress, and directly stimulating root–shoot development, thereby reinforcing soil–plant health linkages during restoration.

### 3.4 Genome-resolved analysis of microbial functional potential

#### 3.4.1 MAG recovery and quality assessment

Metagenomic co-assemblies yielded 44,520 contigs for dung, 11,690 for urine, and 41,308 for *Jeevamrit*, all with consistent assembly quality (N50 ~ 6 kb). Binning recovered 286, 136, and 274 bins for dung, urine, and *Jeevamrit*, respectively. After dereplication, 53 non-redundant MAGs (>50% completeness and <10% contamination) were retained, including 30 met medium- to high-quality thresholds (≥70% completeness, <10% contamination), 14 MAGs satisfying the high-quality MIMAG standards (≥90% completeness, ≤5% contamination). MAG sizes ranged from 1.20–3.42 Mbp, with 60% showing contiguity above 10 kb (N50), and GC content spanning from 37.6% (Fibrobacteres) to 63.9% (Actinobacteria), reflecting phylum-specific genomic traits. (Supplementary Table S2).

**Figure 3:**
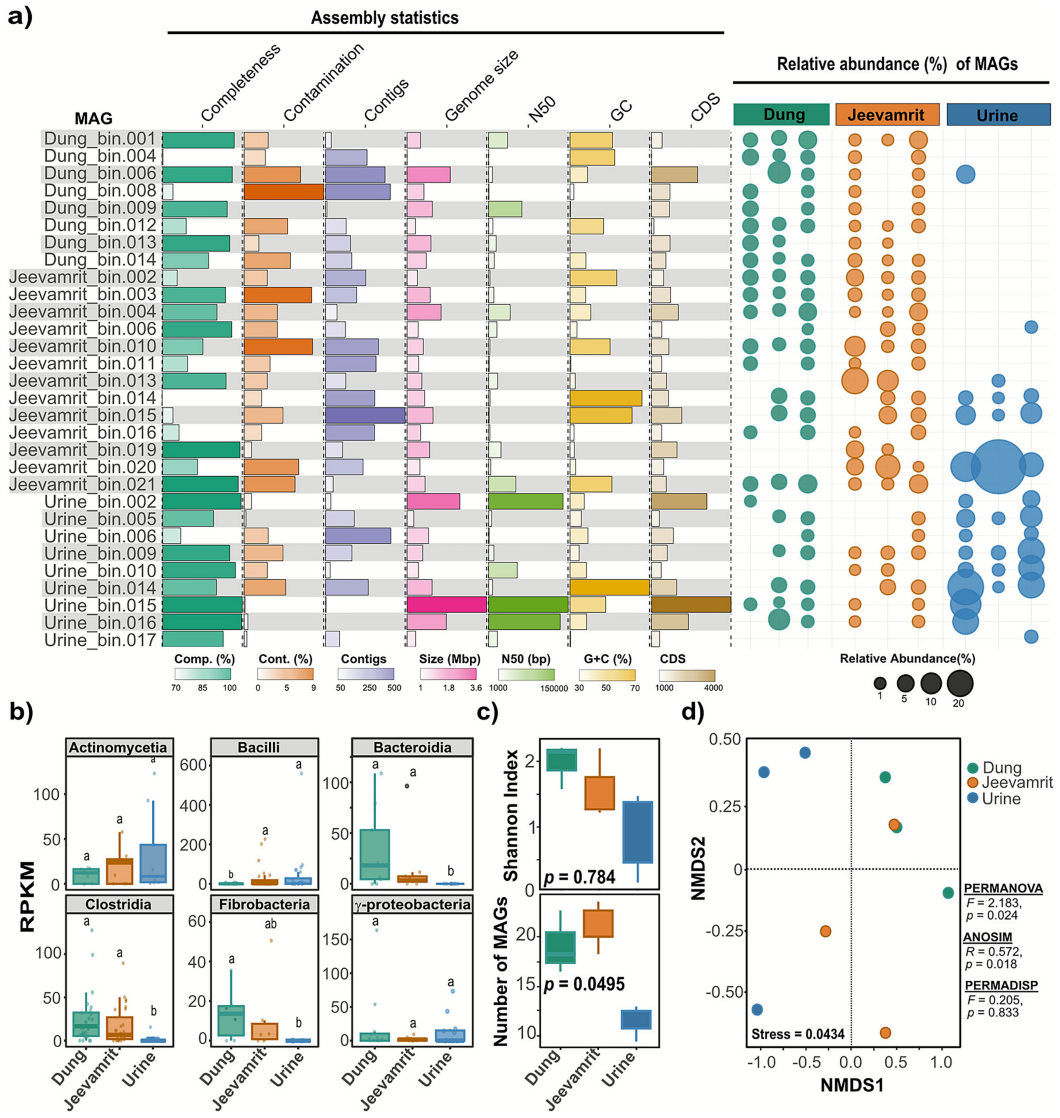
Genome-resolved analysis of microbial community structure across dung, urine, and *Jeevamrit*. **(a)** Assembly statistics and relative abundance of metagenome-assembled genomes (MAGs). Bars show completeness, contamination, contig number, genome size, N50, GC content, and coding sequences (CDS) for representative MAGs recovered from each substrate, with bubble plots on the right indicating their relative abundance. **(b)** Relative abundance (RPKM) of key bacterial classes. Different letters above boxplots indicate significant pairwise differences (*p* < 0.05). **(c)** Boxplots showing alpha diversity (Shannon index and observed number) of MAGs across substrates. **(d)** Non-metric multidimensional scaling (NMDS) ordination of MAG-level community composition based on Bray–Curtis distances, showing distinct clustering of dung, urine, and *Jeevamrit* samples. PERMANOVA, ANOSIM, and PERMDISP results indicate significant substrate-driven differentiation in MAG assemblages.

Relative abundance profiles revealed distinct yet overlapping distributions across substrates (Figure 3a). Dung and *Jeevamrit* hosted richer and more evenly distributed MAG assemblages, whereas urine was dominated by a few highly abundant genomes. Unique MAGs were rare: one in urine (<1%) and one in *Jeevamrit* (~3%), with none in dung. Shared associations were common: dung, *Jeevamrit* shared 13 MAGs, urine–*Jeevamrit* 2 (including one that expanded to dominance in urine), and urine–dung 1. Eleven MAGs were ubiquitous across all three substrates, ranging from rare (<1%) to dominant (>20%). Expressed proportionally, ~3% of MAGs were unique, ~50% shared between two substrates, and ~37% occurred in all three.

Statistical analyses of MAG abundance (RPKM) confirmed differential enrichment of Bacilli (*p-adj* = 0.00403), Bacteroidia (*p-adj* = 0.00403), Clostridia (*p-adj* < 0.0001), and Fibrobacteria (*p-adj* = 0.0431) across substrates (Figure 3b). Despite these compositional differences, alpha diversity analysis indicated comparable richness (Observed: *p-adj* = 0.0495) and evenness (Shannon: *p-adj* = 0.784) across substrates (Figure 3c). NMDS ordination based on Bray–Curtis distances further revealed that urine-derived MAGs clustered distinctly from dung and *Jeevamrit* (PERMANOVA: *F* = 2.183, *R^2^* = 0.4506, *p* = 0.024; Figure 3d), consistent with *Bacilli* enrichment in nitrogen-rich, labile carbon environments.

#### 3.4.2 Taxonomic diversity of the MAGs

**Figure 4:**
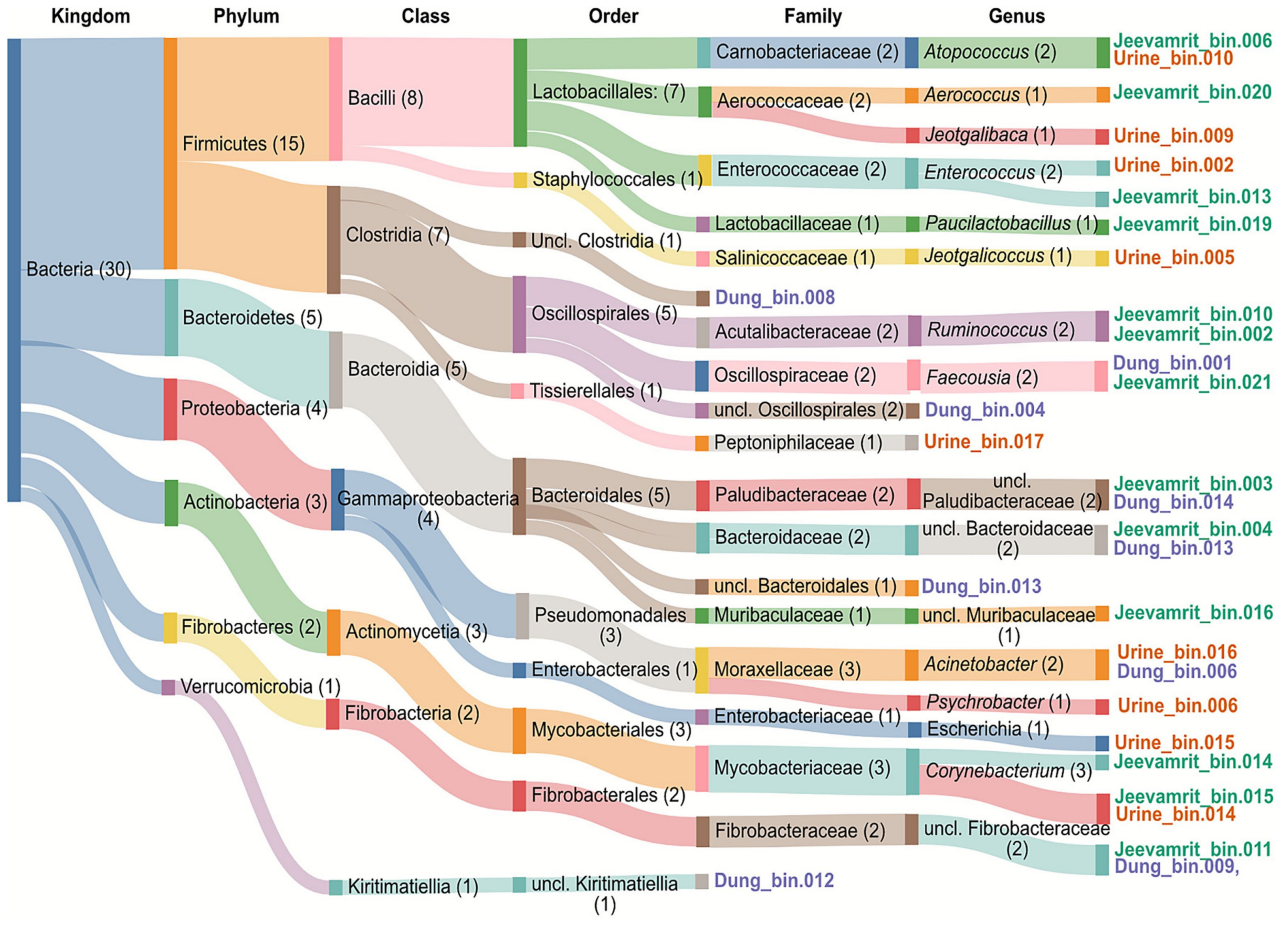
Taxonomic distribution of metagenome-assembled genomes (MAGs) from kingdom to genus level. Hierarchical Sankey diagram illustrating MAG taxonomic assignments across major ranks (Kingdom → Phylum → Class → Order). Flow width is proportional to the number of MAGs assigned to each lineage. Distinct colors represent different phylogenetic lineages, aiding visualization of lineage-specific diversity. Numbers in parentheses indicate MAG count per taxonomic group. The diagram reveals dominance of Firmicutes (*n* = 15 MAGs, 50%), particularly Bacilli (*n* = 8) and Clostridia (*n* = 7), alongside substantial representation of Actinobacteria (*n* = 6) and Bacteroidetes (*n* = 4).

MAG taxonomic profiles revealed clear substrate-linked structuring, with seven bacterial phyla represented and Firmicutes dominating (15 MAGs, ~47%), particularly *Bacilli* and *Clostridia* (Figure 4). At a finer resolution, a clear substrate-specific partitioning of microbial lineages emerged. Urine-derived MAGs included *Jeotgalibaca* (Urine_bin.009) and *Jeotgalicoccus* (Urine_bin.005), while *Jeevamrit* harbored MAGs harbored *Atopococcus* (*Jeevamrit*_bin.006), *Enterococcus* (*Jeevamrit*_bin.013), and *Corynebacterium* (*Jeevamrit*_bin.015), which are associated with multifunctional roles in nitrogen cycling and organic acid production. Dung-derived MAGs clustered with *Faecousia* (Dung_bin.001), *Ruminococcus* (*Jeevamrit*_bin.010), and *Oscillospiraceae* (Dung_bin.001, Dung_bin.004). In addition, unclassified MAGs were recovered within *Oscillospirales* and *Kiritimatiellia* (Figure 4).

#### 3.4.3 MAG-level PGP gene, CAZymes and biosynthetic gene clusters distribution

**Figure 5:**
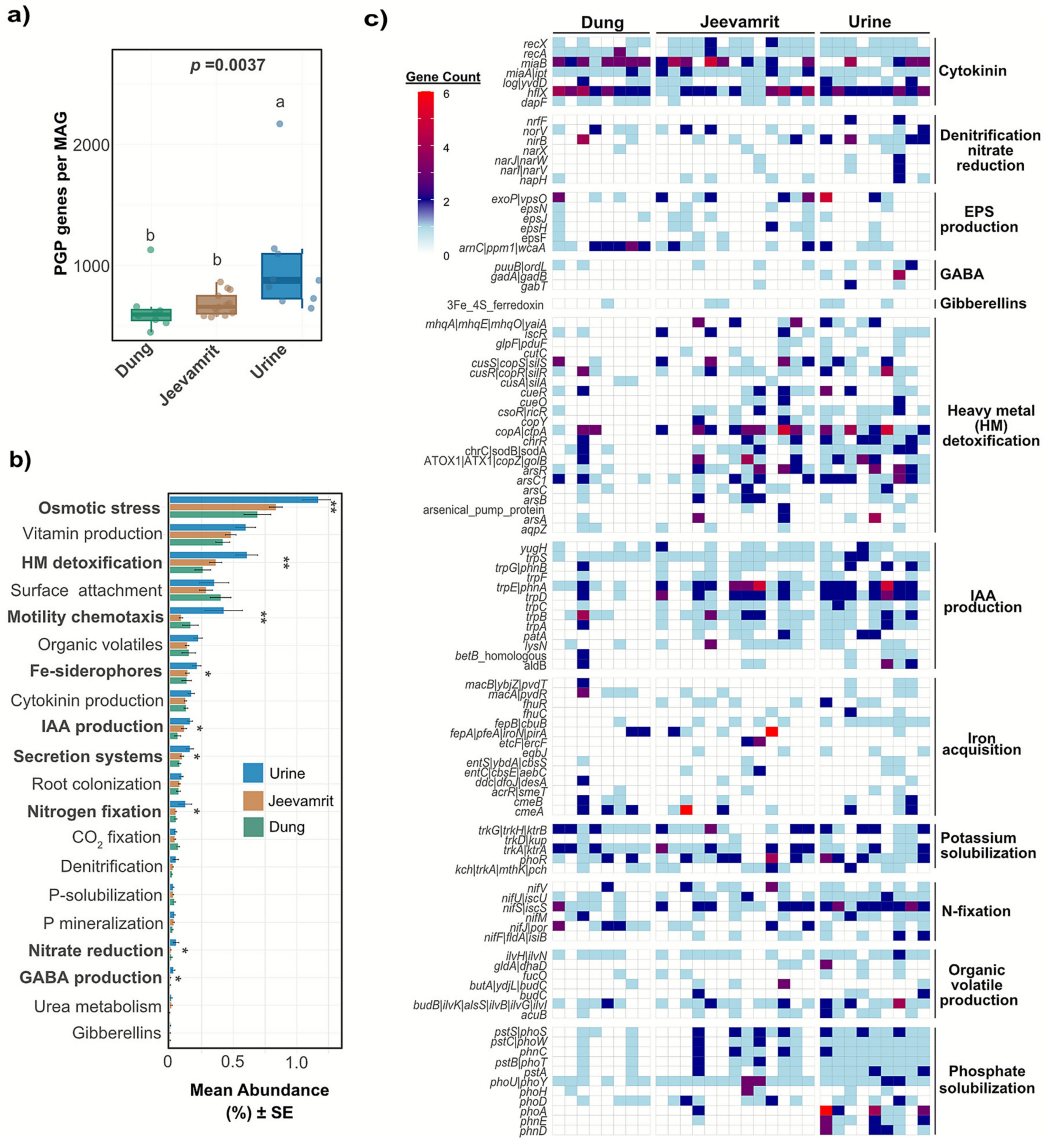
Functional potential of dung, urine, and *Jeevamrit* microbiomes in plant growth promotion (PGP). **(a)** Boxplots showing the number of PGP-associated genes per MAG across substrates. Different letters indicate significant pairwise differences (*p* < 0.05). **(b)** Mean relative abundances (± SE) of major PGP functional categories inferred from metagenomic annotations. Significant differences among substrates are indicated (*p < 0.05, **p < 0.01). **(c)** Heatmap showing distribution of PGP genes categorized according to functional categories across all samples. Gene counts are represented by a red-to-blue gradient scale. Heatmaps are presented as complementary visual summaries of patterns already captured in panels **(a,b)**.

Figure 5 provides a genome-resolved overview of PGP gene distribution across the 30 MAGs, revealing clear substrate-specific enrichment patterns. Urine-derived MAGs carried the highest number of PGP genes (1,006 ± 156; *p* = 0.0037), significantly exceeding those from *Jeevamrit* (678 ± 27) and dung (639 ± 208) (Figure 5a). Functional categories significantly (*p* < 0.05) enriched in urine included osmotic stress tolerance, heavy metal detoxification, motility and chemotaxis, siderophore-mediated iron acquisition, nitrogen fixation, secretion systems, phytohormone production (cytokinin, IAA, GABA), and nitrate reduction (Figure 5b; Supplementary Table S3).

Certain PGP traits were conserved across all MAGs (Figure 5c). Cytokinin biosynthesis genes (rec*X, recA, miaB, miaA|ipt, log|yvdD, hflX*, *dapF*) and potassium solubilization genes (*trkGH|ktrB, trkD|kup, trkA/ktR, phoR*) were nearly ubiquitous. Nitrogen fixation genes showed uneven representation: *nifS/iscS, nifU/iscU,* and *nifJ|por* were widespread, *nifV* was sporadic, while *nifF* was absent from dung MAGs. Phosphate solubilization genes were significantly enriched in urine MAGs (notably *phoU* and *ppk*), whereas *phoH* was scarce across all substrates.

Heavy metal detoxification genes (*copA, sodA, chrR, arsR, arsC1*, ATOX1) were enriched in *Jeevamrit* and urine. Over 60% of *Jeevamrit*-derived MAGs encoded broad combinations of PGP traits, spanning osmotic stress tolerance, siderophore production, nitrogen fixation, secretion systems, and multiple phytohormone pathways. Key multifunctional genomes included *Jeevamrit*_bin.006 (*Atopococcus*), *Jeevamrit*_bin.013 (*Enterococcus*), *Jeevamrit*_bin.015 (*Corynebacterium*), and *Jeevamrit*_bin.020 (*Aerococcus*), each carrying dense repertoires encompassing cytokinin and IAA biosynthesis, phosphate solubilization, and detoxification modules (Figure 5c; Supplementary Table S3).

Comparisons revealed overlap between *Jeevamrit* and urine taxa. Relatives of *Jeevamrit* taxa such as *Atopococcus*, *Corynebacterium*, and *Acinetobacter* were also present in urine-derived MAGs (Urine_bin.010, Urine_bin.014, and Urine_bin.016), showing parallel enrichment in siderophore production, osmotic tolerance, and phosphate solubilization. In contrast, dung-derived MAGs exhibited narrower repertoires, specializing in nutrient-cycling pathways such as partial nitrogenase operons and K^+^ transport, reflecting their gut-origin ecological niches.

**Figure 6:**
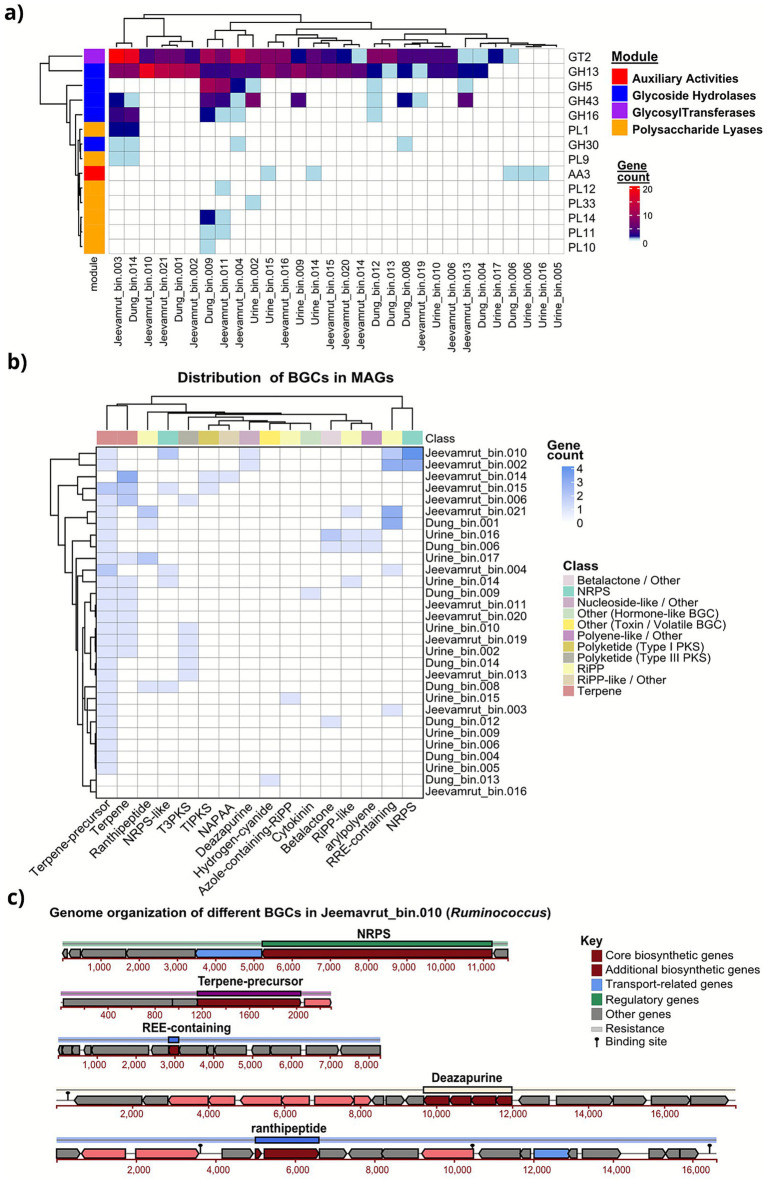
Functional potential for carbohydrate metabolism and secondary metabolite biosynthesis across MAGs from dung, urine, and *Jeevamrit*. **(a)** Heatmap of carbohydrate-active enzyme (CAZyme) families showing substrate-specific enrichment of glycoside hydrolases (GH), glycosyl transferases (GT), polysaccharide lyases (PL), and auxiliary activities (AA). **(b)** Heatmap of biosynthetic gene cluster (BGC) classes across MAGs, highlighting broad metabolic potential in *Jeevamrit*-derived genomes, including nonribosomal peptide synthetases (NRPS), polyketide synthases (PKS), and terpenes. **(c)** Genomic organization of a representative BGC identified in *Jeevamrit*_bin.010 (*Ruminococcus*), showing co-localization of biosynthetic, regulatory, and transporter genes, supporting its role in specialized metabolite production and microbial competitive fitness.

The distinct biochemical environments of cow dung, urine, and *Jeevamrit* were strongly reflected in their CAZyme profiles, with fermentation acting as a key ecological filter (Figure 6a). Our analysis revealed a clear substrate-specific pattern in the distribution of CAZyme genes. Dung-derived MAGs were significantly enriched in glycoside hydrolases (GH13, GH5, GH43), which are critical for the breakdown of complex carbohydrates such as cellulose and hemicellulose. Urine-derived MAGs exhibited the lowest CAZyme diversity, with only six distinct types detected, reflecting the low organic content of urine and providing a baseline for the fermentation process. In contrast, *Jeevamrit* MAGs, showed the highest total CAZyme gene hits (n = 44) and a balanced repertoire of biosynthetic glycosyltransferases (GTs) and degradative GH families. This co-occurrence of biosynthetic (e.g., GT2 for biofilm formation) and degradative (e.g., GH13).

Finally, antiSMASH analysis identified a total of 59 BGCs across metagenome-assembled genomes (MAGs) derived from dung, urine, and the final fermented *Jeevamrit* product (Figure 6b; Supplementary Table S4). These BGCs span 11 functional classes, including NRPS (nonribosomal peptide synthetases), terpenes, and Type III polyketide synthases (T3PKS). MAGs from the final fermented *Jeevamrit* exhibited significantly greater BGC richness and diversity compared to those from raw dung or urine inputs. Taxonomically, these BGCs were predominantly associated with Firmicutes, Actinobacteria, and Bacteroidetes. The widespread detection of terpene and NRPS-like clusters in MAGs affiliated with *Ruminococcus*, *Lactobacillus*, and *Clostridium* indicated potential capacity for producing volatile organic compounds (VOCs) and antimicrobial peptides. Notably, *Jeevamrit*_bin.010, (affiliated with *Ruminococcus*) harbored a diverse biosynthetic repertoire that included ranthipeptides, deazapurine analogs, terpene precursors, NRPS, and RiPP regulatory elements (Figure 6c).

### 3.5 Field trial results

Field validation of *Jeevamrit* across two planting seasons revealed substantial improvements in agronomic and soil health parameters (Table 2).

**Table 2:** Effect of treatments on the yield and soil parameters at harvest of paddy after two planting seasons and sampling.

Treatment	Yield (q ha^−1^)	Soil fertility parameters				Chemical parameters		Biological parameters	
		SOC (%)	Available N (kg/ha)	Available P (kg/ha)	Available K (kg/ha)	pH	EC (dS/m)	MBC (mg C/kg)	DHA (mg TPF/g/h)
T1 (V1-14d)	31.90^a^	1.04^a^	224^a^	32.58^a^	137^a^	5.18	0.10	185.8^b^	118.8^a^
T2 (V1-21d)	29.84^a^	1.04^a^	212^b^	31.37^a^	130^ab^	5.06	0.09	281.5^a^	118.7^a^
T3 (V1-28d)	25.45^ab^	0.76^b^	208^b^	29.54^a^	129^b^	5.94	0.09	276.5^a^	117.6^a^
T4 (V2-14d)	28.67^ab^	0.76^b^	225^a^	31.24^a^	131^ab^	5.04	0.10	201.9^b^	123.2^a^
T5 (V2-21d)	30.23^a^	0.77^b^	210^b^	30.89^a^	128^b^	5.01	0.11	237.3^ab^	117.9^a^
T6 (V2-28d)	27.31^b^	0.77^b^	209^b^	29.55^a^	127^b^	5.02	0.08	218.1^b^	113.9^a^
T7 (V1-Control)	18.33^c^	0.68^c^	208^b^	18.04^c^	114^c^	5.05	0.09	72.22^c^	93.9^b^
T8 (V2-Control)	20.61^c^	0.53^c^	200^c^	23.34^b^	114^c^	4.97	0.09	72.04^c^	94.1^b^
Absolute Δ (95% CI)*	**+8.76 (7.1–10.4)**	**+96.2 (18.9–126.3)**	**+12.4 (8.9–15.9)**	**+9.47 (7.8–11.2)**	**+15.3 (12.1–18.5)**	**–**	**–**	**+159.4 (135.2–183.6)**	**+21.3 (16.8–25.8)**
Cohen’s d	**3.85**	**8.92**	**1.24**	**4.21**	**2.98**			**6.73**	**2.41**

*Mean difference between *Jeevamrit* treatments and controls with 95% confidence intervals. Different superscript letters next to mean values in a columns are significantly different from each other (ANOVA, DMRT, *p* < 0.05). Cohen’s d interpretation: 0.2 = small, 0.5 = medium, 0.8 = large, >1.2 = very large; paddy varieties V1 = HPR-1068; V2 = HPR-2880; Numbers indicate *jeevamrit* application interval in days. Parameters significantly improved from the control treatment are bolded.

#### 3.5.1 Yield performance

*Jeevamrit* treatments achieved significantly higher grain yields compared to controls across both varieties (*F₇,₁₆* = 18.42, *p* < 0.001, effect size = 0.890). For variety HPR-1068 (V1), T1 (14-day interval) produced the highest yield of 31.90 q ha^−1^, followed by T2 (21-day: 29.84 q ha^−1^) and T3 (28-day: 25.45 q ha^−1^), compared to control T7 (18.33 q ha^−1^). Variety HPR-2880 (V2) showed similar trends, with T5 (21-day: 30.23 q ha^−1^) yielding highest, followed by T4 (14-day: 28.67 q ha^−1^) and T6 (28-day: 27.31 q ha^−1^), compared to control T8 (20.61 q ha^−1^). Across all *Jeevamrit* treatments, mean yield was 28.23 q ha^−1^ compared to 19.47 q ha^−1^ in controls, representing an absolute gain of 8.76 q ha^−1^ (95% CI: 7.1–10.4 q ha^−1^; Cohen’s d = 3.85).

#### 3.5.2 Soil organic carbon (SOC) and biological parameters

Soil organic carbon (SOC) exhibited the most pronounced response to Jeevamrit application. V1 treatments T1 (14 d) and T2 (21 d) achieved the highest SOC levels of 1.04%, significantly exceeding all other treatments (*p* ≤ 0.05). V1 treatment T3 (28 d) and all V2 treatments (T4–T6) recorded intermediate SOC values ranging from 0.76 to 0.77%. In contrast, control plots were severely depleted, with SOC levels of 0.68% (T7) and 0.53% (T8). The mean absolute SOC increase relative to controls was 0.96 percentage points (95% CI: 0.53–1.04 pp.; Cohen’s *d* = 8.92), representing a substantial recovery of soil carbon. For context, baseline SOC prior to potato cultivation averaged 0.60% and increased to 1.04% under *Jeevamrit* treatments, reflecting the system’s capacity to restore degraded soils toward levels approaching those found in healthy temperate agricultural systems (4%–6%).

Microbial biomass carbon increased from a baseline of 88.5 mg C kg^−1^ to 185.8–281.5 mg C kg^−1^ across *Jeevamrit* treatments, compared to 72.2 mg C kg^−1^ in controls. The mean absolute gain was 159.4 mg C kg^−1^ (95% CI: 135.2–183.6 mg C kg^−1^; *F*₇,₁*₆* = 98.45, *p* < 0.001). These values represent restoration toward the 200–400 mg C kg^−1^ range typical of biologically active agricultural systems. Similarly, dehydrogenase activity (DHA) ranged from 113.9 to 123.2 mg TPF g^−1^ h^−1^ across all *Jeevamrit* treatments, with no significant differences among them. Controls exhibited significantly lower DHA (T7: 93.9; T8: 94.1 mg TPF g^−1^ h^−1^), representing an average 23.7% increase in *Jeevamrit* treatments.

#### 3.5.3 Nutrient availability and chemical parameters

Available N was highest in T1 and T4 (224–225 kg ha^−1^), significantly exceeding most other treatments and controls (200–212 kg ha^−1^), with an average increase of 12.3% over the controls. Similarly, available P exhibited a consistent enhancement across all *Jeevamrit*-amended soils (29.54–32.58 kg ha^−1^), with T1 recording the maximum value. In contrast, markedly lower P availability was observed in the controls (T7: 18.04; T8: 23.34 kg ha^−1^), reflecting an average improvement of 39.5% under *Jeevamrit* application. Available K also increased significantly in all treated plots (127–137 kg ha^−1^) compared with the controls (114 kg ha^−1^), with T1 again achieving the highest concentration, representing an average gain of 20.2%.

In contrast, soil pH (4.97–5.94) and electrical conductivity (0.08–0.11 dS m^−1^) did not differ significantly among treatments (*p* > 0.05), indicating that *Jeevamrit* application had negligible influence on soil acidity or salinity.

#### 3.5.4 Multivariate analysis

**Figure 7:**
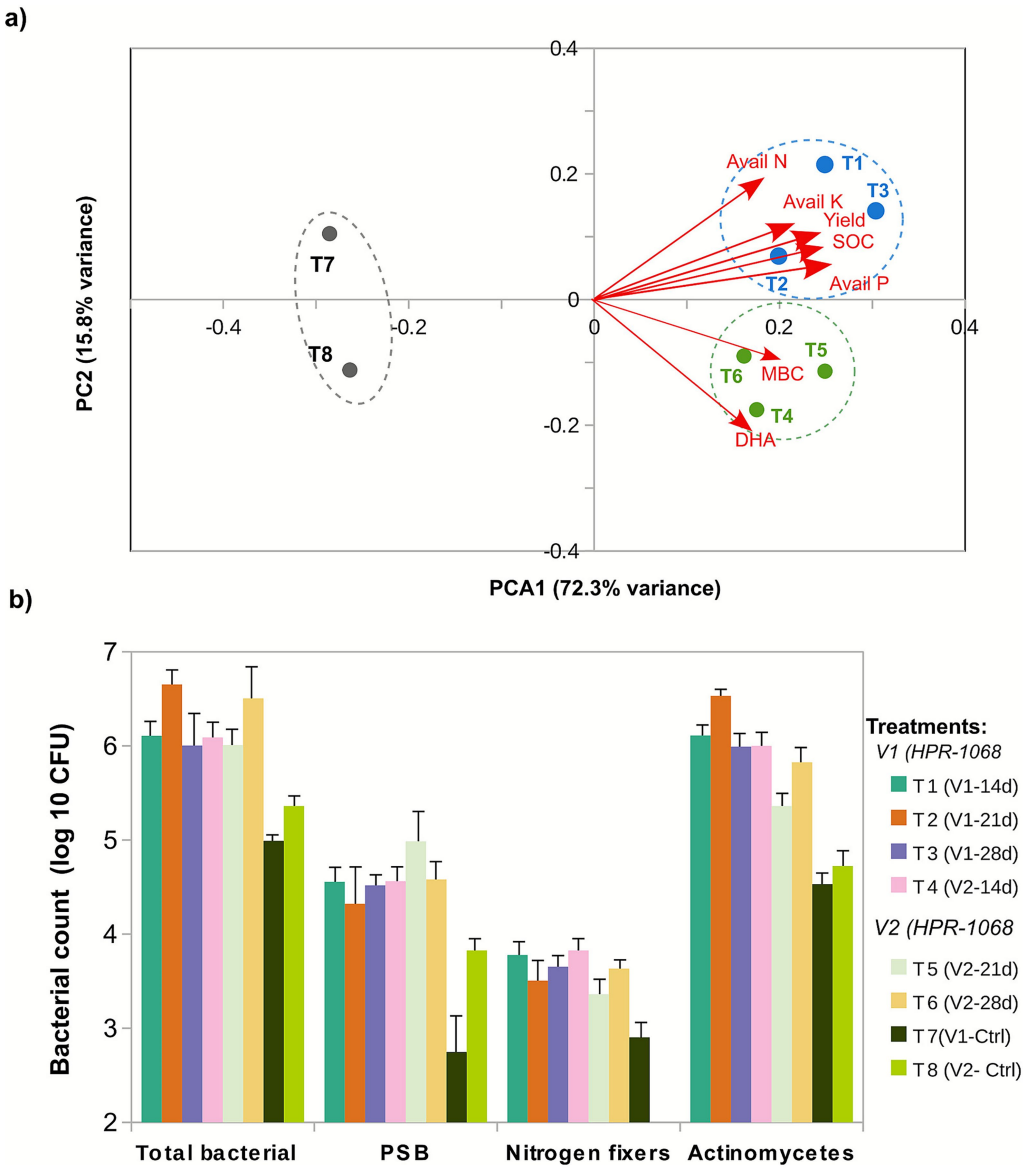
Multivariate assessment of *Jeevamrit* effects on paddy yield and soil restoration parameters. **(a)** Principal component analysis (PCA) biplot showing treatment clustering based on yield and soil parameters after two planting seasons. Data were standardized (*z*-score transformation) prior to analysis to ensure equal weighting of variables measured on different scales. PC1 (72.3% variance) separates *Jeevamrit*-treated plots (right) from controls (left), representing the primary axis of soil health restoration. PC2 (15.8% variance) captures variety-specific responses. Red arrows indicate variable loadings (values shown in parentheses); loadings > |0.40| are considered meaningful contributors. SOC (0.92), MBC (0.90), available *p* (0.89), yield (0.87), and available K (0.82) are primary discriminators along PC1. Ellipses represent 95% confidence intervals for each treatment group. **(b)** Effect of *Jeevamrit* application on soil microbiological health indicators. Data represent means ± standard error of three replicates; bars with different letters are significantly different at *p* ≤ 0.05 according to Duncan’s multiple range test following significant ANOVA (General bacteria: *F₇,₁₆* = 34.2, *p* < 0.001; PSB: *F₇,₁₆* = 28.9, *p* < 0.001; NFB: *F₇,₁₆* = 31.4, *p* < 0.001). V1 = HPR-1068; V2 = HPR-2880; Ctrl = untreated control. PSB = phosphate-solubilizing bacteria; NFB = nitrogen-fixing bacteria.

To elucidate multivariate relationships among soil attributes, principal component analysis (PCA) was performed (Figure 7a). The first two components explained 88.1% of total variance, with PC1 (72.3%) representing the primary axis of soil health differentiation and PC2 (15.8%) capturing variety-specific responses. PC1 clearly separated *Jeevamrit* treatments (positive scores) from controls (negative scores), with all six treatment groups (T1–T6) occupying distinct compositional space from both controls (T7–T8). PC2 further distinguished V1 treatments (positive scores) from V2 treatments (negative scores), producing three well-defined clusters: V1-Jeevamrit, V2-Jeevamrit, and controls. The strongest PC1 contributors were SOC (0.92), MBC (0.90), available *p* (0.89), yield (0.87), and available K (0.82), all projecting in the positive PC1 direction. Their nearly parallel vector orientations indicate high intercorrelation (*r* > 0.94), demonstrating that these soil health indicators respond as an integrated system rather than independently. In contrast, pH and EC (loadings <0.30) showed minimal association with either component, confirming their insensitivity to treatment. Vector length reflects variable importance: the longest vectors (SOC, MBC, available P) exhibited the greatest total variance explained (communalities >0.84; Supplementary Table S5), making them the most reliable indicators of treatment effects. Shorter vectors like DHA (loading = 0.65, communality = 0.61) contributed meaningfully but retained greater unexplained variance, suggesting influence from factors beyond those captured by PC1–PC2.

Furthermore, microbiological enumeration at harvest reinforced the PCA-derived patterns, demonstrating markedly elevated microbial abundance in Jeevamrit-treated soils (Figure 7b). Populations of general bacteria, phosphate-solubilizing bacteria (PSB), and nitrogen-fixing bacteria (NFB) were consistently higher across both rice varieties and all application frequencies compared to the controls. These significant increases (ANOVA, *p* ≤ 0.05) highlight the strong stimulatory effect of *Jeevamrit* on soil biological functioning, indicating that the amendment not only enhances nutrient cycling potential but also supports a more active and resilient microbial community.

## 4 Discussion

This study provides genome-to-field evidence that *Jeevamrit* functions as a multifunctional microbial consortium capable of restoring biological fertility, nutrient availability, and crop productivity in degraded soils. By integrating metagenomic, genome-resolved, and field validation analyses, we demonstrate that fermentation of cattle-derived substrates generates a metabolically diverse microbial community with strong plant growth–promoting (PGP) and biogeochemical potential. These findings advance mechanistic understanding of how farmer-developed fermented organic fertilizers (FOFs) such as *Jeevamrit* mediate soil rejuvenation through synergistic microbial interactions rather than through nutrient supplementation alone.

The integration of metagenomic signatures with field data revealed a clear mechanistic continuum linking functional gene enrichment to measurable improvements in soil fertility and yield. Fermentation transformed the microbiota of dung and urine into a stable consortium enriched in diazotrophs, phosphate-solubilizers, and organic matter decomposers, guilds central to nutrient cycling and soil aggregation (Bargaz et al., 2018; Compant et al., 2019; Chakraborty et al., 2025). The recovery of 30 high-quality MAGs spanning Firmicutes, Actinobacteria, and Bacteroidetes illustrates the emergence of a functionally redundant microbial network capable of sustaining soil biological processes under low-input conditions. IIn the field, these genomic potentials translated into marked improvements relative to degraded controls, including SOC increases of 52%–96% (0.53%–1.04%), MBC gains of 159–281 mg C kg^−1^, and grain yield enhancements of 45% (8.76 q ha^−1^) (Table 2), confirming that *Jeevamrit* functions as a biological catalyst for soil system recovery. To elucidate the microbial mechanisms underlying these improvements, we examined functional gene enrichments across key nutrient cycles.

### 4.1 Mechanisms of nutrient enhancement

The enrichment of *nif*, *nas*, and *nir* gene families in *Jeevamrit* metagenomes highlights the central role of microbial nitrogen cycling in fertility restoration. Nitrogenase subunits (*nifH*, *nifD*, *nifK*) and assimilatory nitrate reduction genes (*nasA*, *nasB*, *nirA*) were strongly represented (Figures 2, 5), indicating both biological N₂ fixation and nitrate assimilation capacities. These functions were taxonomically linked to *Bradyrhizobium elkanii*, *Bacillus megaterium*, and *Streptomyces* spp., known diazotrophs with facultative anaerobic metabolisms suited to microaerophilic soil niches (Wang et al., 2023). The presence of these lineages supports sustained nitrogen turnover even under oxygen fluctuations typical of rice paddy soils. Field data corroborated these genomic predictions: available N increased by 12.3% over controls, especially in T1 and T4 (224–225 kg ha^−1^). This improvement likely reflects a dual mechanism, direct nitrogen fixation by introduced microbes and stimulation of indigenous N-transforming populations through organic carbon inputs. The coexistence of urease and nitrate reductase pathways suggests balanced ammonification and nitrification, which can mitigate N losses through volatilization or leaching (Ren et al., 2023). Thus, *Jeevamrit* fosters a microbially mediated N balance, sustaining plant-available nitrogen while reducing dependency on synthetic fertilizers.

In terms of P metabolism, *Jeevamrit*-amended soils exhibited a 39.5% increase in available phosphorus, consistent with the genomic enrichment of *pstS*, *phoA*, and *phoB* genes encoding high-affinity phosphate transporters and alkaline phosphatases. Such genes facilitate P acquisition under limiting conditions by releasing orthophosphates from insoluble forms (Alori et al., 2017). The predominance of *Bacillus*, *Pseudomonas*, and *Corynebacterium* MAGs carrying these traits aligns with earlier reports that these genera dominate the rhizosphere of organic systems and contribute to P mobilization through organic acid secretion and phosphatase production (Alori et al., 2017; Mukherjee et al., 2025). These microbial and genomic signatures align with agronomic findings that *Jeevamrit* enhances soil phosphorus availability and plant uptake, particularly when integrated with amendments like vermitea or rock phosphate (Gurjar et al., 2024). Additionally, the co-occurrence of siderophore biosynthetic genes (*fepA*, *fhuA*, *feoB*) (Figures 2, 5) suggests indirect mechanisms of P solubilization via Fe chelation and mineral weathering (Goff et al., 2023; Kümmerli, 2023). For instance*, fepA* and *feoB* mediate Fe^3+^ and Fe^2+^ uptake, respectively, while *exbB* facilitates siderophore transport, enhancing microbial competitiveness and plant iron nutrition under limiting conditions (Lai, 2021)(Lai, 2021). These processes release bound phosphates from Fe/Al complexes in acidic soils (Nannipieri et al., 2011; Alori et al., 2017). The observed improvement in available P across all treatments therefore reflects a functionally layered microbial process, integrating direct solubilization, transporter-mediated uptake, and chelation-assisted mobilization.

Similarly, all *Jeevamrit* treatments showed significant increases in available K (127–137 kg ha^−1^ versus 114 kg ha^−1^ in controls). At the mechanistic level, the enrichment of *trkA*, *trkG*, and *phoR* genes, coupled with actinomycete-associated organic acid production, supports enhanced K solubilization from aluminosilicates. Actinomycetes and *Bacillus* spp. release carboxylic acids and siderophores that weather K-bearing minerals, contributing to the sustained release of exchangeable K (Das and Pradhan, 2016). The balanced enrichment of these microbial guilds indicates that *Jeevamrit* not only replenishes immediate nutrient pools but also reactivates the mineral nutrient economy of degraded soils.

Comparative metagenomic analysis highlights two features that distinguish *Jeevamrit* from other fermented organic fertilizers, particularly bokashi (Abo-Sido et al., 2021) and Andes biol (O’Neill and Ramos-Abensur, 2022). First, *Jeevamrit* possesses a broader nitrogen cycling repertoire: while bokashi is enriched mainly in ammonification genes (e.g., *ureABC, gdhA*) with limited capacity for N fixation, *Jeevamrit* contains complete *nifHDK* operons across multiple taxa. This enables both N recycling and *de novo* N inputs, overcoming the substrate-dependent limitations of lactic acid bacteria–dominated systems. Second, P mobilization in *Jeevamrit* is more diverse and persistent. Unlike bokashi, which relies largely on transient organic acid–driven solubilization (Abo-Sido et al., 2021), *Jeevamrit* integrates phosphatases, high-affinity transporters, and siderophore-mediated mineral weathering, supporting longer-term P availability. Field evidence, particularly the sustained 39.5% increase in available P, corroborates the genomic signals. Together, these results position *Jeevamrit* as a biologically dynamic amendment capable of long-term integration into soil nutrient cycles, surpassing conventional fermented organic fertilizers whose effects are primarily substrate driven.

### 4.2 Soil restoration capacity of *Jeevamrit* treatments

SOC improvement (up to 1.04%) represents one of the most profound indicators of biological recovery. Genome-level enrichment of carbohydrate-active enzymes (CAZymes), particularly glycoside hydrolases (GH13, GH43) and polysaccharide lyases (PL1, PL9) (Figure 7a), underscores *Jeevamrit*’s capacity for controlled organic matter decomposition. These enzymes break down complex polysaccharides into assimilable substrates, supporting microbial proliferation and humic substance formation (Lladó et al., 2017). Concurrently, glycosyltransferases (GT2, GT4) involved in extracellular polysaccharide (EPS) synthesis promote carbon stabilization via soil aggregation and protection of organic carbon within microaggregates (Zethof et al., 2020). This dual role of decomposition and stabilization helps explain the observed SOC gains from 0.53%–0.68% in controls to 0.76%–1.04% in treated plots, alongside the correlated increase in microbial biomass carbon (MBC), which represents the dynamic carbon pool driving humification and long-term soil carbon sequestration (Lal, 2015).

The substantial increase in MBC (up to 281.5 mg C kg^−1^) and dehydrogenase activity (DHA; up to 123.2 mg TPF/g/h, 23%–26% above controls) indicates reactivation of microbial metabolism. MBC serves as an early indicator of nutrient cycling capacity, while DHA reflects intracellular oxidation–reduction activity linked to microbial energy transfer (Nannipieri et al., 2011). These biochemical responses likely arise from synergistic enrichment of microbial guilds possessing siderophore, phytohormone, and stress-tolerance genes, as seen in MAGs of *Atopococcus*, *Corynebacterium*, and *Enterococcus*. Increased MBC enhances enzymatic turnover of nutrients, closing feedback loops that sustain soil fertility even under low external input. The strong correlation among SOC, MBC, and yield (PCA loadings >0.85) further supports a causal linkage between microbial metabolic activation and agronomic performance (Figure 7; Supplementary Table S5). The multivariate analysis confirmed that *Jeevamrit’*s restoration effects operate through coordinated enhancement of multiple soil properties rather than isolated parameter improvements, supporting the functional redundancy model wherein overlapping microbial metabolic capacities drive integrated soil system recovery.

To place our findings in practical context, we compared *Jeevamrit*-restored soil parameters against regional benchmarks for the Himalayan mid-hills and global standards for productive agricultural systems. The Indian Council of Agricultural Research (ICAR) classifies SOC levels as: very low (<0.40%), low (0.40%–0.75%), medium (0.75%–1.00%), and high (>1.00%) for acidic Himalayan soils (Babu et al., 2020; Mourya et al., 2021). In our study, control plots exhibited SOC values of 0.53%–0.68%, placing them at the upper range of the “low” category and reflecting the degraded status typical of potato monoculture systems (Lepcha and Devi, 2020). *Jeevamrit* application substantially increased SOC to 0.76%–1.04%, elevating soils into the “medium to high” range and surpassing conventional thresholds for effective organic matter restoration. These gains demonstrate that *Jeevamrit* not only replenishes carbon stocks in depleted soils but also drives them toward levels associated with sustainable nutrient cycling and long-term productivity, approaching the SOC concentrations (1.2–1.8%) reported in well-managed temperate rice-based systems (Lal, 2015). Similarly, MBC values <100 mg C kg^−1^ indicate severely impaired biological function (Gao et al., 2022), while values >200 mg C kg^−1^ characterize biologically active systems. Control plots (72 mg C kg^−1^) confirmed severe biological degradation, whereas *Jeevamrit* treatments (186–282 mg C kg^−1^) restored microbial activity to or above functional thresholds (Table 2) (Lepcha and Devi, 2020). Available P increased from 18–23 kg ha^−1^ (low to medium range per ICAR guidelines) to 30–33 kg ha^−1^ (medium to high range), reflecting improved nutrient cycling capacity (Veerabhadrappa et al., 2024; Renshu et al., 2025). Yield gains (28.2 vs. 19.5 q ha^−1^) must be contextualized within the severely degraded baseline. The regional average rice yield for organic systems in Himachal Pradesh is 35–40 q ha^−1^, suggesting that *Jeevamrit* restored productivity to 70%–80% of regional organic potential from a degraded starting point of 50%–55% (Eyhorn et al., 2018; Babu et al., 2020).

### 4.3 Microbial community assembly and functional redundancy

Fermentation acted as an ecological filter, assembling synergistic guilds from dung and urine into a resilient *Jeevamrit* community. *Jeevamrit* retained dung-associated Firmicutes and Bacteroidetes but integrated urine-derived Proteobacteria, enriching both metabolic diversity and functional redundancy. Such redundancy, multiple taxa performing overlapping ecological roles, enhances resilience under environmental fluctuations (Allison and Martiny, 2008). PCA of field data mirrored this genomic assembly pattern: *Jeevamrit* treatments clustered distinctly from controls, driven by SOC, MBC, available P, and yield. These findings suggest that soil microbial networks were not only replenished but reprogrammed toward higher functional capacity and stability. The recurrent dominance of *Bacillus*, *Ruminococcus*, and *Corynebacterium* MAGs across both genomic and field datasets underscores their roles as keystone taxa, organic matter turnover, rhizosphere colonization, and multi-pathway nutrient transformations.

Unlike EM solutions and defined synthetic consortia, which often suffer ecological incompatibility in field conditions due to narrow phylogenetic composition and limited metabolic flexibility (Prisa and Jamal, 2025), *Jeevamrit* consists of a broad, reproducible assemblage of 30 MAGs spanning Firmicutes, Actinobacteria, Proteobacteria, and Bacteroidetes. These MAGs carried overlapping functional capacities across nitrogen cycling, phosphorus mobilization, osmotic stress adaptation, and redox tolerance (Figure 5c), providing a multidimensional redundancy network that aligns with known predictors of microbial resilience (Allison and Martiny, 2008). Beta-diversity analysis demonstrated a stable community structure across fermentation batches (PERMANOVA, *p* = 0.61), highlighting reproducibility despite biological substrate variability.

The detection of 59 BGCs across 30 *Jeevamrit* MAGs, encoding NRPS, PKS, terpene, and RiPP pathways (Figures 6b,c), indicates substantial biosynthetic capacity for secondary metabolite production. However, several caveats warrant emphasis before attributing field outcomes to these genetic elements. First, BGC presence does not guarantee expression: transcriptomic studies show that 30%–60% of BGCs remain silent under standard growth conditions (Amos et al., 2017). Without corresponding metabolomic validation, our predictions remain hypothetical functional potential rather than confirmed activity. Factorial experiments with sterile *Jeevamrit* filtrates and metabolomic tracking in rhizosphere exudates would be necessary to distinguish direct BGC-mediated effects from indirect substrate-driven processes. Second, we benchmarked our findings against null expectations to assess genuine enrichment. The 1.97 BGCs per MAG in *Jeevamrit* modestly exceeds random soil community averages (0.8–1.2 per MAG) but falls below specialized Actinobacteria-enriched systems (2.4–3.1 per MAG) (Crits-Christoph et al., 2018). However, our observed BGC class diversity (11 functional types across 30 genomes) exceeds typical soil surveys (8–12 classes across 100 + genomes), suggesting selective enrichment of biosynthetically versatile taxa during fermentation rather than random community assembly. Third, functional annotation revealed that 39% of BGCs encode antimicrobial or siderophore biosynthesis pathways (vs. 15–20% in bulk soils), potentially contributing to enhanced rhizosphere competitiveness. This functional skew represents a notable advancement over previously characterized bokashi and FOF systems, which show limited secondary metabolite biosynthetic capacity (Abo-Sido et al., 2021; Chakraborty et al., 2025).

Notably, the *Ruminococcus* MAG (Jeevamrit_bin.010) harbored a diverse biosynthetic arsenal including ranthipeptides, NRPS, PKS, and terpene precursors (Figure 6c). While *Ruminococcus* spp. are typically characterized as fibrolytic anaerobes, recent genomic surveys document underappreciated biosynthetic capacity in this clade (Anderson and Fernando, 2021), supporting our hypothesis that cattle gut-derived taxa contribute specialized metabolites facilitating soil colonization. Similarly, *Lactobacillus*-associated terpene clusters suggest potential for VOC-mediated plant signaling, influencing growth promotion and defense priming (Wu et al., 2022; Jaffar et al., 2023). NRPS- and PKS-derived metabolites theoretically enhance competitive fitness through pathogen suppression (antimicrobial peptides) and micronutrient scavenging (siderophores) (Grosse et al., 2023; Zelaya-Molina et al., 2025). Complementary phytohormone biosynthesis genes (*trpA, miaB, speE*) indicate additional routes for modulating root development, cell division, and stress tolerance. These integrated functional capacities help explain the sustained increases in microbial biomass alongside the absence of disease pressure, ultimately supporting the yield gains observed in *Jeevamrit* treatments (up to 31.9 q ha^−1^ in T1) (Table 2).

Critically, however, we cannot exclude alternative explanations. Substrate priming effects from jaggery and pulse flour inputs could stimulate indigenous microbes independently of introduced BGC-bearing taxa. The observed field improvements may reflect synergistic interactions among (i) direct antimicrobial activity from expressed BGCs, (ii) enhanced nutrient cycling from primary metabolic genes, and (iii) physical and chemical conditioning from organic substrate inputs. Disentangling these contributions will require controlled experiments tracking individual mechanisms. Nevertheless, the combination of demonstrated BGC class diversity (11 types), functional skew toward competitive metabolites (39% antimicrobial/siderophore), and integration with plant growth–promoting traits positions *Jeevamrit* as a potentially self-regulating system. Its capacity to simultaneously enhance nutrient availability, maintain microbial stability, and theoretically suppress opportunistic pathogens distinguishes it from substrate-dependent or compositionally narrow biofertilizer systems, though metabolomic validation remains essential to confirm these hypothesized mechanisms.

A critical challenge in biofertilizer research is distinguishing *introduced microbial activity* from *stimulation of indigenous communities*. Metagenomic tracking studies using strain-specific markers (not performed in our study) suggest that 40%–70% of introduced taxa fail to establish beyond 30–60 days post-application (Sukhum et al., 2021). Our detection of Jeevamrit-associated taxa (*Bacillus, Ruminococcus, Corynebacterium*) at harvest (120 days post-final application) via enumeration suggests successful colonization, but we cannot exclude the possibility that indigenous strains harboring similar functional genes were selectively enriched rather than introduced taxa persisting. Future studies employing genome-resolved metagenomics of treated soils would resolve this ambiguity.

### 4.4 Field validation and agronomic implications

The field trials provided empirical validation of *Jeevamrit’*s multi-tiered microbial effects. The significant yield gains and nutrient restoration observed across two crop varieties indicate that *Jeevamrit*’s benefits are robust across genotypic contexts. To contextualize our findings within the broader biofertilizer literature, we compared *Jeevamrit’s* performance against recent meta-analyses and systematic reviews. Fan et al. (2023) conducted a global meta-analysis of 124 field trials evaluating organic fertilizers, reporting mean yield increases of 3.48% for tomatoes when replacing 100% of synthetic fertilizers. Our 45% yield gain (8.76 q ha^−1^ absolute increase) in zero-synthetic-input systems exceeds this benchmark, positioning *Jeevamrit* in the upper quartile of FOF efficacy. However, direct comparisons are complicated by differences in baseline soil fertility: our control SOC (0.53%–0.68%) was substantially lower than meta-analysis means (1.2%–1.8%), suggesting that degradation severity amplifies relative treatment effects. Treatments with 14–21-day application intervals consistently outperformed longer intervals, suggesting that microbial activity peaks within 3 weeks of application, aligning with the typical lifespan of actively fermenting microbial consortia. Importantly, soil pH and electrical conductivity remained stable (*p* > 0.05), demonstrating that *Jeevamrit* enhances fertility without altering soil chemical balance, a key attribute for sustainable use in acidic Himalayan soils. Collectively, the congruence between genomic potential and field performance confirms that *Jeevamrit* acts through biological intensification rather than chemical supplementation. Its ability to regenerate microbial biomass, recycle nutrients, and stabilize organic matter makes it a scalable strategy for restoring productivity in nutrient-depleted agroecosystems.

While the genomic–agronomic alignment is compelling, it is important to note that the field component relied on three biological replicates per treatment, the standard for plot-scale agronomic trials but insufficient for structural equation modeling (SEM) or regression-based causal analysis. Such approaches typically require substantially larger sample sizes to support model stability and avoid overfitting. Consequently, our analysis focuses on effect sizes and correlations, which are the statistically appropriate methods for low-replicate experimental designs. We explicitly acknowledge this limitation and emphasize that future multi-site trials with higher replication, including intermediate temporal sampling, will allow formal causal modeling linking MAG abundances to agronomic outcomes while also capturing seasonal and inter-annual variability.

This work situates *Jeevamrit* within the broader paradigm of microbiome-based soil restoration and agroecological intensification (Lal, 2015; Compant et al., 2019). The mechanistic distinctions discussed above position *Jeevamrit* not merely as an alternative FOF but as a functionally differentiated system whose cow-urine-dung co-fermentation strategy assembles a metabolically versatile, ecologically resilient microbial network. Zero-input controls (our design) amplify treatment effects relative to reduced-fertilizer comparisons. Studies comparing *Jeevamrit* + 50% synthetic NPK vs. 100% synthetic NPK report more modest 8%–15% yield advantages (Saharan et al., 2023), suggesting *Jeevamrit* functions optimally as a replacement rather than supplement strategy. The integration of MAG-level functional genomics with two-season field validation provides a replicable framework for mechanistically validating other traditional FOFs, addressing the evidence gap that has excluded such systems from climate finance mechanisms and regulatory approval pathways despite widespread farmer adoption. By demonstrating direct linkages between metagenomic function and agronomic outcomes, it provides a framework for functional validation of biofertilizers that bridges the gap between laboratory predictions and field realities. The identification of key PGP taxa and gene markers also offers a basis for developing standardized microbial quality indicators, addressing variability that currently limits FOF reliability (Mukherjee et al., 2025).

Overall, our findings position *Jeevamrit* as a microbial ecosystem engineer, a living formulation that re-establishes functional soil microbiomes, enhances nutrient cycling, and restores productivity in degraded soils. Its multifunctionality exemplifies how traditional knowledge systems, when examined through modern genome-resolved frameworks, can yield scientifically validated, sustainable solutions for climate-resilient agriculture. In summary, our findings align with meta-analytical evidence that fermented consortia deliver substantial soil restoration benefits in degraded systems, while exceeding typical effect sizes due to extreme baseline depletion. The genomic characterization adds mechanistic depth absent from most meta-analyzed studies, which rely on culture-dependent or amplicon-based methods with limited functional resolution.

## 5 Conclusion

This study provides the first genome-to-field mechanistic validation of *Jeevamrit,* demonstrating functional and ecological differentiation from existing fermented biofertilizer systems, while acknowledging inherent complexities in attributing field outcomes to specific microbial processes. Unlike lactic acid bacteria-dominated bokashi or defined EM consortia, *Jeevamrit’s* co-fermentation of cattle dung and urine assembles a functionally redundant microbial network enriched in both nitrogen fixation (*nifHDK*) and phosphate solubilization (*phoA, pstS*) gene clusters, conferring dual nutrient regeneration and mobilization capacities. Genome-resolved metagenomic analyses revealed 30 high-quality MAGs spanning Firmicutes, Actinobacteria, and Bacteroidetes, encoding synergistic pathways for nitrogen cycling, organic matter turnover, biosynthetic secondary metabolite production, siderophore biosynthesis, and phytohormone production. Our data support a functional redundancy model wherein soil restoration stems from overlapping metabolic capacities distributed across multiple taxa rather than single keystone species or taxonomic diversity per se. These genomic potentials translated into substantial field-level gains such as increased soil organic carbon, microbial biomass carbon, available phosphorus, and grain yield, while maintaining pH and EC stability. The results demonstrate that *Jeevamrit* promotes microbial ecological intensification, rejuvenating soil fertility through active biological processes rather than chemical supplementation. By bridging traditional agroecological knowledge with modern microbial genomics, this work establishes a mechanistic framework for validating farmer-innovated biofertilizers as standardized, scalable soil restoration technologies. While BGC enrichment suggests enhanced biosynthetic potential for rhizosphere colonization and pathogen suppression, metabolomic validation remains necessary to confirm realized secondary metabolite production. Future research should employ isotope tracing, sterile filtrate controls, and genome-resolved metagenomics of treated soils to disentangle introduced microbial activity from substrate-mediated priming of indigenous communities, ultimately strengthening causal attribution. *Jeevamrit* exemplifies a living microbial ecosystem capable of regenerating soil health in severely degraded systems, offering a replicable model for scaling nature-based, low-cost agricultural technologies to address global sustainability challenges and contribute to climate-resilient food production.

## Data Availability

Libraries were prepared using the Twist NGS Library Preparation Kit (Twist Biosciences, USA) and quality-checked on an Agilent 2100 Bioanalyzer. Sequencing was conducted on the Illumina NovaSeq 6000 platform (2 × 150 bp). All raw sequence data have been deposited in the NCBI Sequence Read Archive under BioProject ID PRJNA1162843, with individual SRA accessions SRR30724627 to SRR30724635.

## Footnotes

1
https://www.kbase.us

2
https://doi.org/10.6084/M9.FIGSHARE.29894891

3
https://github.com/wwood/CoverM
