# Supplementary material for: Genome-resolved analysis of traditional fermented biofertilizers as scalable solutions for soil restoration
Source: Front Microbiol. 2025 Dec 29;16:1725475. doi: 10.3389/fmicb.2025.1725475 (PMC12791043; doi:10.3389/fmicb.2025.1725475)
Supplement: Supplementary file 2 [file Image_1.pdf]

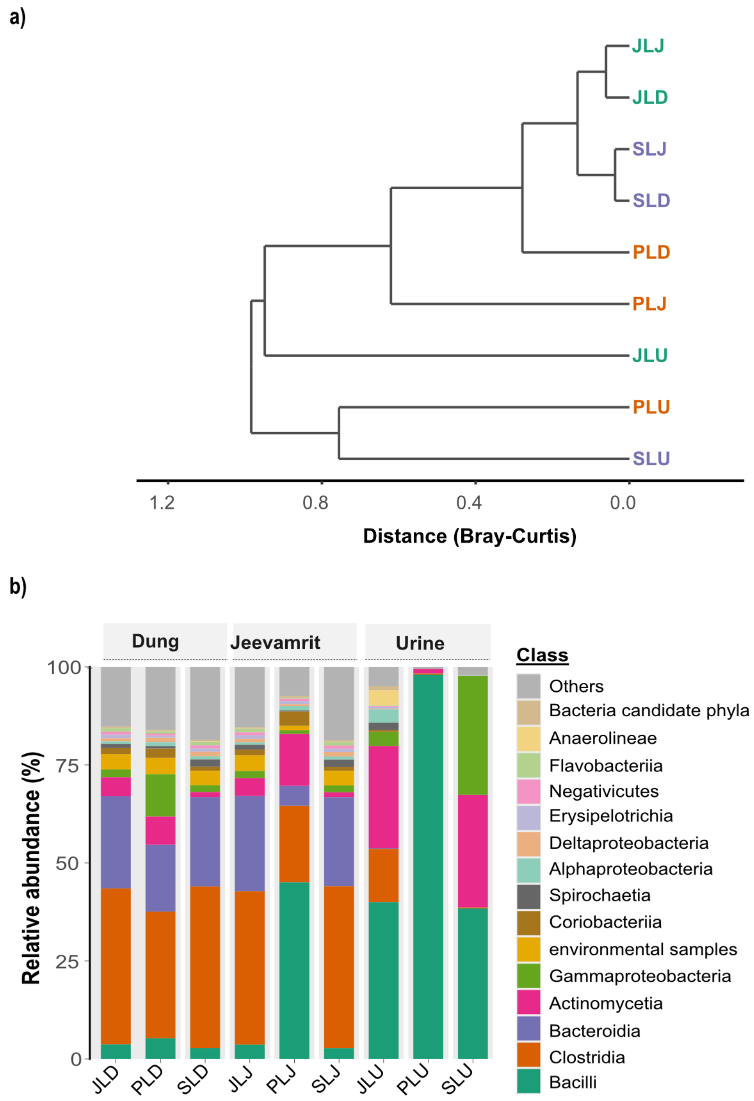

Figure S1. Substrate-driven microbial community assembly in *Jeevamrit* (a) Hierarchical clustering dendrogram of Bray–Curtis dissimilarities showing clear partitioning of dung, urine, and *Jeevamrit* microbiomes, confirming substrate-specific community structures. (b) Relative abundances of dominant bacterial classes across substrates, highlighting functional guild partitioning: dung enriched in anaerobic fermenters (Clostridia, Bacteroidia), urine dominated by nitrogen-processing Proteobacteria, and *Jeevamrit* integrating both guilds into a functionally complementary consortium.
